# A multi-strategy enhanced RIME-based metaheuristic with adaptive strategy collaboration for global optimization and art image segmentation

**DOI:** 10.1038/s41598-026-49814-3

**Published:** 2026-05-18

**Authors:** Xianmeng Zhao, Yutong Duan, Fan Liu

**Affiliations:** School of Innovation and Design, Wuhan Textile University, Wuhan, 430070 Hubei Province China

**Keywords:** RIME optimization algorithm, Image segmentation, Heuristic algorithm, Differential evolution operation, Global optimization, Engineering, Mathematics and computing

## Abstract

Traditional Rime Optimization Algorithm (RIME) often encounters performance degradation when dealing with complex optimization landscapes, such as high dimensionality, strong multimodality, and application-driven image segmentation tasks. To overcome these limitations, this study develops a multi-strategy self-adaptive Rime Optimization approach, termed MSRIME, which enhances both search diversity and convergence stability through adaptive and collaborative mechanisms. Specifically, MSRIME introduces a dynamically adjusted differential mutation factor to regulate exploration and exploitation along the optimization process. In addition, a heterogeneous strategy pool composed of multiple update operators is constructed, enabling complementary search behaviors at different optimization stages. A probability-driven strategy selection scheme based on performance feedback is further employed to adaptively allocate search resources among strategies. The proposed algorithm is evaluated on the CEC2017 and CEC2022 benchmark suites and compared with several state-of-the-art metaheuristic algorithms. Experimental results show that MSRIME consistently achieves superior performance in terms of optimization accuracy and robustness. In the Friedman statistical test, MSRIME obtains the best mean rankings of 1.93 and 1.77 on CEC2017 (30D and 100D), and 1.67 and 2.17 on CEC2022 (10D and 20D), respectively, outperforming all competing algorithms. Furthermore, MSRIME is applied to multilevel threshold image segmentation using Otsu’s criterion. The results demonstrate that the proposed algorithm achieves higher PSNR, SSIM, and FSIM values across different images and threshold levels, indicating its effectiveness in practical applications. Overall, the proposed MSRIME provides an effective and robust optimization framework, achieving significant performance improvements without increasing computational complexity.

## Introduction

In many scientific and engineering domains, complex optimization problems are ubiquitous, including structural design^1^, path planning^2,3^, feature selection^4^, image processing^5^, machine learning model training^6^, medical diagnosis^7^, and industrial scheduling. As problem scales continue to grow and structures become increasingly intricate, numerous optimization tasks exhibit characteristics such as nonlinearity, multimodality, high dimensionality, non-differentiability, and strong noise disturbances. These properties often render traditional analytical methods or gradient-based deterministic algorithms ineffective in finding satisfactory solutions^8–10^. Consequently, the development of optimization methods that offer efficient search capability, strong robustness, and broad applicability has become one of the central research directions in contemporary intelligent computing.

Optimization algorithms play a crucial role in real-world applications, including medical image analysis, engineering design, intelligent manufacturing, and machine learning. In particular, high-quality optimization techniques are essential for solving complex problems such as feature selection, parameter tuning, and image segmentation, where traditional deterministic methods often fail^11–14^.

Over the past two decades, intelligent optimization algorithms have attracted extensive attention due to their gradient-free nature, ease of implementation, and strong adaptability to complex search spaces. These algorithms are typically inspired by certain natural phenomena or mechanisms, designing search strategies by simulating biological evolution, animal swarm cooperation, physical and chemical processes, or human social behaviors^15,16^. According to their sources of inspiration, existing intelligent optimization algorithms can be broadly categorized into the following groups: evolutionary algorithms (EA), swarm intelligence (SI) algorithms, physics- and chemistry-based optimization algorithms, and social- or cognitive-inspired algorithms^6,17^.

Evolutionary algorithms are grounded in Darwin’s theory of evolution and update the population through the mechanisms of “selection–crossover–mutation.” Representative methods include Genetic Algorithm (GA)^18^, which simulates biological reproduction to maintain population diversity and global search capability, is one of the earliest and most classical evolutionary algorithms. Differential Evolution (DE)^19^, which relies on differential mutation strategies, is favored for its simple structure, strong optimization ability, and stable global performance. Evolution Strategy (ES)^20^ emphasizes the self-adaptive adjustment of step sizes and strategy parameters, providing strong local exploitation capabilities in certain continuous optimization problems. Overall, evolutionary algorithms offer wide search coverage and strong robustness. However, their limitations include relatively slow convergence speed, multiple hyperparameters, and a need for careful parameter tuning.

Swarm intelligence algorithms realize population-based search by simulating cooperation and information sharing among individuals in animal groups. Representative methods such as Particle Swarm Optimization (PSO)^21^ algorithm performs efficient search through velocity and position updates, though it is prone to premature convergence.

Ant Colony Optimization (ACO)^22^, inspired by the foraging behavior of ant colonies, and the Grey Wolf Optimizer (GWO)^23^, which simulates wolf-pack encircling and hunting behaviors, both feature simple structures and strong exploitation capabilities. Furthermore, algorithms such as the Whale Optimization Algorithm (WOA)^24^, Seagull Optimization Algorithm (SOA)^25^, Harris Hawks Optimization (HHO)^26^, Butterfly Optimization Algorithm (BOA)^27^, Sled Dog Optimizer (SDO)^28^, Secretary Bird Optimization Algorithm (SBOA)^29^, Cuckoo Catfish Optimizer (CCO)^30^, Cape Lynx Optimizer (CLO)^31^, Bounty Hunter Optimizer (BHO)^32^, and Dung Beetle Optimization (DBO)^33^ implement information sharing and dynamic search through various animal-inspired mechanisms. Implement information sharing and dynamic search through various animal-inspired mechanisms.

Optimization algorithms based on physical and mathematical mechanisms construct their search frameworks by simulating energy transitions, dynamic equilibria, or material transformation processes in physical systems. Representative examples Simulated Annealing (SA)^34^, which models the thermodynamic annealing process to escape local optima; Gravitational Search Algorithm (GSA)^35^, which simulates gravitational attraction to achieve global interaction among agents; as well as the Arithmetic Optimization Algorithm (AOA)^36^, Kirchhoff’s Law Algorithm (KLA)^37^, and others. Although these algorithms offer stronger theoretical interpretability, they often suffer from relatively complex structures and challenging parameter control.

Algorithms inspired by social and cognitive behavior design optimization procedures based on human society, cooperation, and learning processes. Typical examples include the Teaching–Learning-Based Optimization (TLBO) algorithm^38^, which simulates the teacher–student interaction to perform two-phase updates, and the Great Wall Construction Algorithm (GWCA)^39^, which draws inspiration from the competition and elimination mechanisms observed during ancient Great Wall construction. These algorithms feature few parameters and simple implementation but tend to have limited exploitation capability, making it difficult to maintain long-term optimization effectiveness. To better illustrate the characteristics and limitations of existing metaheuristic algorithms, a comparative summary is provided in Table 1, which highlights the necessity of developing more effective optimization strategies.

**Table 1:** Summary of representative metaheuristic algorithms.

Algorithm	Year	Main idea	Advantages	Limitations
GA^18^	1975	Evolutionary selection	Strong global search	Slow convergence
PSO^21^	1995	Velocity-position update	Fast convergence	Premature convergence
DE^19^	1997	Differential mutation	Simple & effective	Sensitive to parameter
SA^34^	1983	Thermal annealing process	Escapes local optima	Slow convergence
ACO^22^	1996	Pheromone-based search	Good path finding ability	Slow convergence, stagnation
GWO^23^	2014	Wolf hunting mechanism	Strong exploitation	Weak exploration
WOA^24^	2016	Whale bubble-net feeding	Simple, few parameters	Easily trapped locally
HHO^26^	2019	Hawk hunting strategy	Balanced search	Instability in late stage
BOA^27^	2019	Fragrance-based search	Good adaptability	Poor local refinement
DBO^33^	2022	Dung beetle behavior	Strong exploration	Convergence speed varies
AOA^36^	2021	Arithmetic operators	Simple structure	Limited exploitation
TLBO^38^	2011	Teacher–student learning	Few parameters	Weak exploitation
RIME^40^	2023	Biochemical phenomenon of rime ice	Simple, flexible	Weak diversity & exploration

In recent years, multi-strategy enhancement has become an important research direction in metaheuristic optimization. By integrating multiple search operators or adaptive mechanisms, such approaches aim to balance exploration and exploitation more effectively. For example, hybrid strategies combining differential evolution, adaptive parameter control, and dynamic operator selection have demonstrated superior performance in solving complex optimization problems. Recent studies^41–43^ have shown that multi-strategy frameworks can significantly improve convergence speed, solution accuracy, and robustness by dynamically coordinating heterogeneous search behaviors. These works highlight that incorporating multiple complementary strategies is an effective way to overcome the limitations of single-mechanism algorithms.

Multilevel threshold image segmentation, as a crucial component of image preprocessing, plays an irreplaceable role in medical image diagnosis (e.g., tumor region localization)^44^, remote sensing image interpretation (e.g., land-cover classification), and industrial quality inspection (e.g., defect detection). Its core objective is to accurately partition grayscale intervals to maximize the separability between the target and the background^45,46^. As application scenarios grow increasingly complex, traditional optimization methods such as gradient-based approaches and genetic algorithms have gradually shown limitations, including poor adaptability and low optimization efficiency. This situation calls for the development of more efficient and robust intelligent optimization algorithms to address these challenges.

Among the algorithms proposed in recent years, the Rime Optimization Algorithm (RIME)^40^ has attracted increasing attention due to its mathematical simplicity, flexible update mechanism, and low parameter dependency. Its core idea is to drive population evolution through a stochastic input model. Compared with many classical metaheuristic algorithms, the Rime Optimization Algorithm (RIME) exhibits several distinctive advantages, including a simple mathematical structure, low parameter dependency, and a flexible update mechanism driven by stochastic input modeling. These characteristics make RIME particularly suitable as a foundational framework for further enhancement. Moreover, unlike many recently proposed algorithms that rely heavily on multiple control parameters or complex hybrid mechanisms, RIME provides a relatively clean and extensible structure, which facilitates the integration of adaptive strategies without significantly increasing algorithmic complexity. However, existing studies indicate that RIME still suffers from several limitations: insufficient global exploration when tackling complex multimodal functions, relatively slow convergence speed, rapid loss of population diversity in the middle and later stages, and a single update paradigm that lacks multi-source collaborative search mechanisms^47,48^.

Since its introduction, the RIME has attracted increasing attention, and several improved variants have been proposed to enhance its performance. Existing studies mainly focus on hybridization with other metaheuristics, adaptive parameter tuning, and diversity preservation mechanisms. For instance, recent RIME variants have introduced chaotic mapping, adaptive control parameters, and hybrid search strategies to improve global exploration and avoid premature convergence^49–51^. Although these variants have achieved certain improvements, most of them still rely on single or loosely coupled strategies and lack a unified multi-strategy collaborative framework. This limitation motivates the development of a more systematic multi-strategy enhancement approach, as proposed in this paper.

Moreover, in multilevel threshold image segmentation tasks, threshold optimization methods based on traditional intelligent algorithms (such as PSO and GWO) often lead to blurred segmentation boundaries due to inadequate optimization accuracy or produce inconsistent segmentation results across multiple runs due to poor stability. These shortcomings make it difficult for such methods to meet the stringent segmentation quality requirements of application scenarios such as medical imaging and remote sensing image analysis.

To address these issues, this paper proposes a Multi-Strategy Enhanced Rime Optimization Algorithm (MSRIME), which integrates multi-source guidance, cooperative population search, random perturbation, and diversity-preservation mechanisms into the RIME framework, thereby enhancing global exploration, local exploitation, and overall stability from multiple dimensions. The main contributions of this study are as follows:

1. MSRIME Algorithm Design: An adaptive differential mutation factor adjustment strategy is introduced to dynamically tune the DE mutation factor F according to the iteration stage, achieving a balance between exploration and exploitation. A collaborative update pool containing four differentiated strategies is constructed, and a probability-based adaptive strategy selection mechanism is designed. This mechanism quantifies the effectiveness of each strategy via a performance counter and dynamically adjusts selection probabilities using a roulette-wheel scheme, improving the algorithm’s adaptability to different problem types.
2. Global Optimization Performance Validation: MSRIME is evaluated on the CEC2017 and CEC2022 benchmark suites and compared with the original RIME and eight mainstream intelligent optimization algorithms. The assessment focuses on optimization accuracy, stability, and convergence efficiency, demonstrating the statistical superiority of MSRIME.
3. Multilevel Threshold Image Segmentation Validation: Using Otsu’s maximum between-class variance criterion as the objective function, 4-, 6-, 8-, and 10-level segmentation experiments are conducted on six benchmark images of different styles. Peak Signal-to-Noise Ratio (PSNR), Structural Similarity (SSIM), and Feature Similarity (FSIM) are used as evaluation metrics to compare the segmentation quality of MSRIME with other algorithms, verifying its practical effective-ness in real-world image processing tasks.

The remainder of this paper is organized as follows: Chapter 2 presents the fundamental principles of the RIME algorithm and details the core enhancements of MSRIME; Chapter 3 evaluates the performance of MSRIME on global optimization problems through numerical experiments; Chapter 4 conducts multilevel threshold image segmentation experiments and analyzes the algorithm’s practical effectiveness; Chapter 5 concludes the study, highlights current limitations, and discusses potential directions for future research.

## RIME optimization algorithm and the proposed MSRIME

### RIME optimization algorithm (RIME)

The Rime Optimization Algorithm (RIME), proposed in 2023, is an innovative optimization method inspired by the natural growth mechanism of rime formations. The algorithm primarily consists of four core steps: (1) rime cluster initialization; (2) soft rime search strategy, simulating the motion characteristics of soft rime particles; (3) hard rime piercing mechanism, which enhances exploration by mimicking the crossover behavior of hard rime particles; and (4) forward greedy selection mechanism. The following provides a detailed mathematical description^40,52^.

(1) Rime Cluster Initialization

Based on the observed natural phenomenon, each agent in the algorithm is treated as a core search entity, and the entire set of agents constitutes the complete rime population. First, the rime population $R$ is constructed by randomly generating $N$ search agents $S_{i}$ in the search space, where each rime agent contains $D$ rime particles $r_{ij}$. The mathematical expression is given by Eq. (1). Therefore, the rime population is described by the rime particles $R_{ij}$, and the initialization process can be expressed as follows^40,52^:

1 $$\begin{array}{*{20}c} {R_{i} \left( j \right) = LB\left( j \right) + rand \times \left( {UB\left( j \right) - LB\left( j \right)} \right)} \\ \end{array}$$

where $i$ denotes the index of the rime agent $\left( {i \in \left\{ {1,2, \ldots ,D} \right\}} \right)$, $j$ denotes the index of the rime particle $\left( {j \in \left\{ {1,2, \ldots ,D} \right\}} \right)$, $rand$ is a random number in the interval [0,1], and $UB$ and $LB$ represent the upper and lower bounds of the search space, respectively.

(2) Soft Rime Search Strategy

In windy environments, the formation of soft rime exhibits significant randomness. It can attach to different surfaces while primarily growing in a single direction. This randomness forms the core of the proposed soft rime search strategy, which leverages the diverse growth patterns of rime particles to enhance search space coverage during the early stages of optimization. The growth of soft rime is mathematically expressed as follows^40,52^:

2 $$\begin{array}{*{20}c} {R_{i}^{{\text{new }}} \left( j \right) = \left\{ {\begin{array}{*{20}l} {R_{{\text{best }}} \left( j \right) + F_{{\text{actor }}} \times \left( {R_{i} \left( j \right) - R_{{\text{best }}} \left( j \right)} \right) \times h,} \hfill & {\quad if\;r_{2} < E} \hfill \\ {R_{i} \left( j \right), } \hfill & {\quad {\mathrm{otherwise}}} \hfill \\ \end{array} } \right.} \\ \end{array}$$

where $R_{ij}^{{\text{new }}}$ denotes the updated position of the *i*th particle of the *i*th rime agent; $R_{{{\text{best }}j}}$ represents the *j*th particle of the globally best rime agent in the population; $F_{{\text{actor }}}$ is a multiplicative factor calculated by Eq. (3); $h$ represents the adhesion coefficient, a random number in the interval (0,1); $r_{2}$ is a random number in (0,1); and $E$ is the adhesion factor influencing condensation probability, computed by Eq. (4)^40,52^:

3 $$\begin{aligned} F_{{\text{actor }}} & = r_{1} \times {\mathrm{cos}}\theta \times \beta \\ \theta & = \frac{2\pi \times t}{{T_{max} }} \\ \beta & = 2 \times \exp \left( { - \left( {\frac{4 \times t}{{T_{max} }}} \right)^{2} } \right) \\ \end{aligned}$$

4 $$\begin{array}{*{20}c} {E = \left\lfloor {\frac{t}{{T_{max} \times W}}} \right\rfloor \times \frac{W}{{T_{max} }}} \\ \end{array}$$

where $r_{1}$ is a parameter controlling the particle movement direction, randomly selected from [−1, 1], $t$ is the current iteration number, $T_{max}$ is the maximum number of iterations, $\left\lfloor \cdot \right\rfloor$ denotes the floor operation, and $W$ with a default value of 5, controls the number of segments in the step function.

(3) Hard Rime Piercing Mechanism

Hard rime grows strictly along a single direction, which can easily lead to overlap among rime agents. Based on this characteristic, a hard rime piercing mechanism is designed to enhance interactions between search agents within the rime population. This mechanism aims to improve the convergence speed of RIME while reducing the risk of trapping in local optima. Its mathematical expression is given as follows^40,52^:

5 $$\begin{array}{*{20}c} {R_{i}^{{\text{new }}} \left( j \right) = R_{{{\mathrm{best}}}} \left( j \right) + F_{{\text{norme }}} \left( {S_{i} } \right) \times r_{3} \times \left( {R_{i} \left( j \right) - R_{{\text{best }}} \left( j \right)} \right)} \\ \end{array}$$

where $F_{{\text{norme }}} \left( {S_{i} } \right)$ denotes the normalized fitness value of the *i*th search agent, reflecting the probability of the agent being selected; $r_{3}$ is a randomly generated number in the interval [−1,1].

(4) Forward Greedy Selection Mechanism

In traditional greedy selection mechanisms, only the globally best agent and the population’s best fitness are updated at each iteration. This approach primarily serves as a record and does not actively drive the population toward effective search, limiting further exploration and exploitation. To address this limitation, RIME introduces a forward greedy selection mechanism to improve search efficiency^40,52^.

In this improved mechanism, the fitness of each updated search agent is compared with its previous fitness. If the updated fitness is better, the corresponding original agent and its fitness are replaced, forming an optimized pair of search agents. Through this proactive replacement strategy, the population continuously retains high-quality agents, thereby improving the overall quality of search agents and guiding the population toward better solutions at each iteration.

### Multi-strategy Self-adaptive Rime Optimization Algorithm (MSRIME)

#### Adaptive differential mutation factor adjustment strategy

In the standard RIME algorithm, the search mechanism relies on fixed parameters during iterations. Core parameters, such as the Rime factor (Rime Factor), vary according to a preset schedule with the iteration count, lacking adaptive capability to the dynamic optimization process. In the early stage of optimization, fixed parameters are insufficient to support broad global exploration, which may lead the algorithm to become trapped in local regions. In the later stage, they cannot facilitate fine exploitation of the optimal solution region, resulting in inadequate convergence precision.

To address this limitation, MSRIME introduces an adaptive differential mutation factor adjustment strategy, which dynamically tunes the mutation factor $F$ in the differential evolution (DE) component, achieving an adaptive balance between exploration and exploitation.

The core logic of this strategy is as follows: based on the iteration progress, a linearly dynamic adjustment model for the mutation factor $F$ is constructed. In the early stage, a relatively large mutation factor is used to strengthen global exploration; as iterations proceed, the mutation factor gradually decreases, guiding the algorithm toward local fine-tuning. Its mathematical expression is:

6 $$\begin{array}{*{20}c} {F = F_{min} + \left( {F_{max} - F_{min} } \right) \times \frac{{T_{max} - t}}{{T_{max} }}} \\ \end{array}$$

where $F_{min} = 0.5$ and $F_{max} = 1$ denote the minimum and maximum values of the mutation factor, respectively.

Through the dynamic adaptation of the mutation factor, the algorithm can traverse the search space with larger steps during the early stage, effectively avoiding limitations of the initial search region. In the later stage, smaller steps focus on the vicinity of the optimal solution, enhancing convergence precision. Importantly, this strategy achieves adaptive tuning solely based on iteration progress, without introducing additional complex environment-sensing parameters, balancing effectiveness and simplicity.

#### Construction of multi-strategy collaborative update pool

The standard RIME algorithm relies on a single “soft rime search+hard rime piercing” update mechanism, which suffers from limited strategy adaptability. On one hand, a single strategy cannot meet the requirements of the full optimization cycle: the early stage requires strong exploration to cover a wide solution space, but the standard RIME’s exploration mechanism overly depends on guidance from the best agent and lacks sufficient population diversity; the later stage requires strong exploitation to refine the optimal solution, yet its local search mechanism lacks efficient information exchange, leading to an imbalance between exploration and exploitation. On the other hand, a fixed strategy struggles to adapt to different types of optimization problems, and for high-dimensional or multimodal problems, it easily encounters convergence stagnation.

To address these limitations, MSRIME constructs a strategy pool containing four differentiated update strategies, covering the entire optimization cycle through collaborative multi-strategy coordination. The strategies are designed as follows:

**Strategy 1: Standard RIME Native Update Strategy (Retention and Adaptation)**

This strategy serves as the baseline, retaining the core logic of the standard RIME, and ensures fundamental optimization capability for low-dimensional, unimodal problems.

**Strategy 2: DE/rand/1 Differential Evolution Strategy (Enhanced Global Exploration)**

This strategy randomly selects three distinct agents to construct a differential vector, enhancing population diversity. It is suitable for the early stage of optimization and global exploration. Its update rule is:

7 $$\begin{array}{*{20}c} {R_{i}^{{\text{new }}} \left( j \right) = \left\{ {\begin{array}{*{20}c} {R_{r1} \left( j \right) + F \times \left( {R_{r2} \left( j \right) - R_{r3} \left( j \right)} \right), if {\text{rand }} < CR} \\ {R_{i} \left( j \right), {\mathrm{otherwise}}} \\ \end{array} } \right.} \\ \end{array}$$

where $R_{r1} ,R_{r2} ,R_{r3}$ are three randomly selected distinct agents $\left( {r1,r2,r3 \ne i} \right)$, $CR = 0.95$, is the crossover probability, and $F$ is the adaptive mutation factor calculated as in Section “Adaptive differential mutation factor adjustment strategy”.

**Strategy 3: DE/current-to-best/1 Differential Evolution Strategy (Balanced Exploration and Exploitation)**

This strategy combines information from the current agent and the global best agent, guiding the agent toward the optimal solution region using a differential vector. It is suitable for the mid-stage of optimization. Its update rule is:

8 $$\begin{array}{*{20}c} {R_{i}^{{\text{new }}} \left( j \right) = \left\{ {\begin{array}{*{20}l} {R_{i} \left( j \right) + F \times \left( {R_{{\text{best }}} \left( j \right) - R_{i} \left( j \right) + R_{r1} \left( j \right) - R_{r2} \left( j \right)} \right),} \hfill & {\quad if\;{\text{rand }} < CR} \hfill \\ {R_{i} \left( j \right),} \hfill & {\quad {\mathrm{otherwise}}} \hfill \\ \end{array} } \right.} \\ \end{array}$$

where $R_{r1} ,R_{r2}$ are two randomly selected distinct agents $\left( {r1,r2 \ne i} \right)$. By introducing information from the global best agent, this strategy accelerates convergence while maintaining population diversity.

**Strategy 4: Random-Weighted Differential Strategy (Enhanced Local Exploitation)**

This strategy introduces a random weight coefficient $L$ to strengthen fine search around the optimal solution, suitable for the late stage of optimization. Its update rule is:

9 $$\begin{array}{*{20}c} {R_{i}^{{\text{new }}} \left( j \right) = R_{i} \left( j \right) + L \times \left( {R_{{\text{best }}} \left( j \right) - R_{i} \left( j \right) + R_{r1} \left( j \right) - R_{r2} \left( j \right)} \right)} \\ \end{array}$$

where $L = rand$ is a random weight in [0,1], and $R_{r1} ,R_{r2}$ are two randomly selected distinct agents $\left( {r1,r2 \ne i} \right)$. Through dynamic adjustment of the random weight, this strategy enables adaptive fine search around the optimal solution, improving convergence precision.

#### Strategy probability adaptive selection mechanism

The single-strategy update mode of the standard RIME lacks dynamic adjustment capability and cannot switch or adapt strategies based on the optimization progress or the population state, resulting in insufficient robustness for complex problems. MSRIME introduces a strategy probability adaptive selection mechanism based on the logic of “strategy performance feedback—probability dynamic adjustment,” enabling intelligent adaptation to the optimization process.

The core logic of this mechanism is divided into three stages:

(1) Quantitative Evaluation of Strategy Performance

In each iteration, a performance counter $c\left( s \right)$ is assigned to each strategy ($s = 1,2,3,4$ corresponding to the four strategies in the strategy pool). For an agent updated using strategy $s$, if the updated fitness satisfies $f\left( {R_{ij}^{{\text{new }}} } \right) < f\left( {R_{ij} } \right)$ (i.e., fitness improvement), the counter of strategy $s$ is incremented by 1, expressed as:

10 $$\begin{array}{*{20}c} {c\left( s \right) = c\left( s \right) + 1,iff\left( {R_{ij}^{new} } \right) < f\left( {R_{ij} } \right)} \\ \end{array}$$

The effectiveness of each strategy in the current iteration is quantified using its performance counter $c\left( s \right)$.

(2) Dynamic Update of Strategy Selection Probability

Based on the performance counters, the selection probability $p\left( s \right)$ of each strategy is calculated. If the total number of successful updates of all strategies $\sum\nolimits_{s = 1}^{4} {c\left( s \right) > 0}$, the probability is positively correlated with the strategy’s performance, expressed as:

11 $$\begin{array}{*{20}c} {p\left( s \right) = \frac{c\left( s \right)}{{\mathop \sum \nolimits_{s = 1}^{4} c\left( s \right)}}} \\ \end{array}$$

(3) Roulette Wheel Strategy Selection

A roulette wheel algorithm is employed to realize stochastic selection of strategies. First, the cumulative distribution of strategy probabilities is computed as: ${\text{cum\_p(s) }} = \sum\nolimits_{k = 1}^{s} {p\left( k \right)}$. Then, a random number [0,1] is generated, and the strategy $s$ satisfying ${\text{cum\_p }}\left( {s - 1} \right) < r \le {\text{ cum\_p }}\left( s \right)$ (${\text{cum\_p }}\left( 0 \right) = 0$ is selected as the update strategy for the current agent.

The advantage of this mechanism lies in its dynamic adaptation of strategy selection to the optimization process: in the early stage, exploration-oriented strategies (Strategy 2) have higher selection probabilities due to more frequent successes, supporting global exploration; in the mid-stage, balanced strategies (Strategy 3) gain higher probabilities, enabling a transition from exploration to exploitation; in the late stage, exploitation-oriented strategies (Strategy 4) dominate, enhancing fine local search. Through performance-feedback-driven probability adjustment, the algorithm significantly improves adaptability across different optimization phases and problem types.

**Fig. 1:**
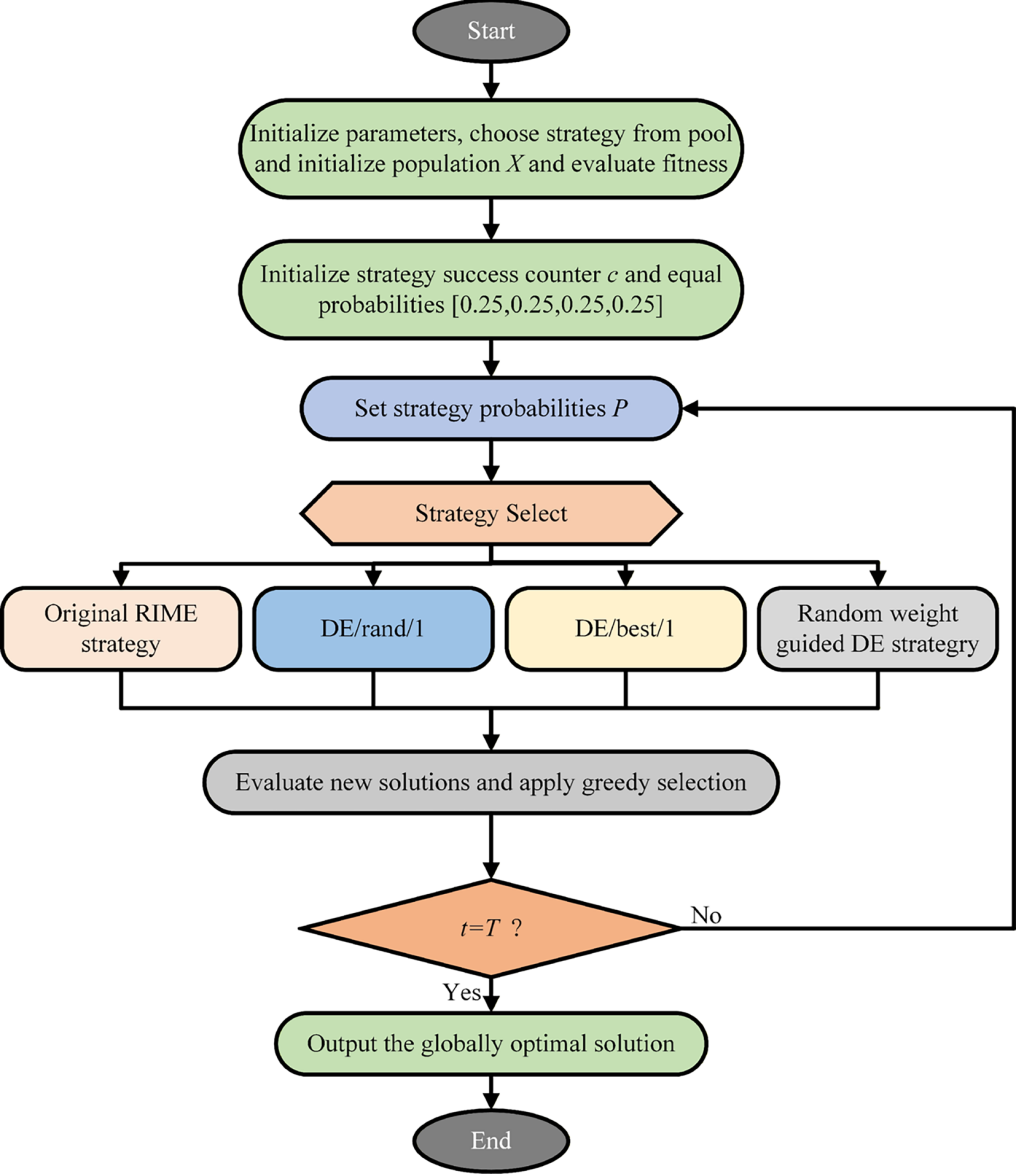
The overall workflow of the proposed MSRIME algorithm.

Algorithm 1 contains the pseudocode pertaining to MSRIME and the overall workflow of the proposed MSRIME algorithm is illustrated in Fig. 1.

**Algorithm 1:**
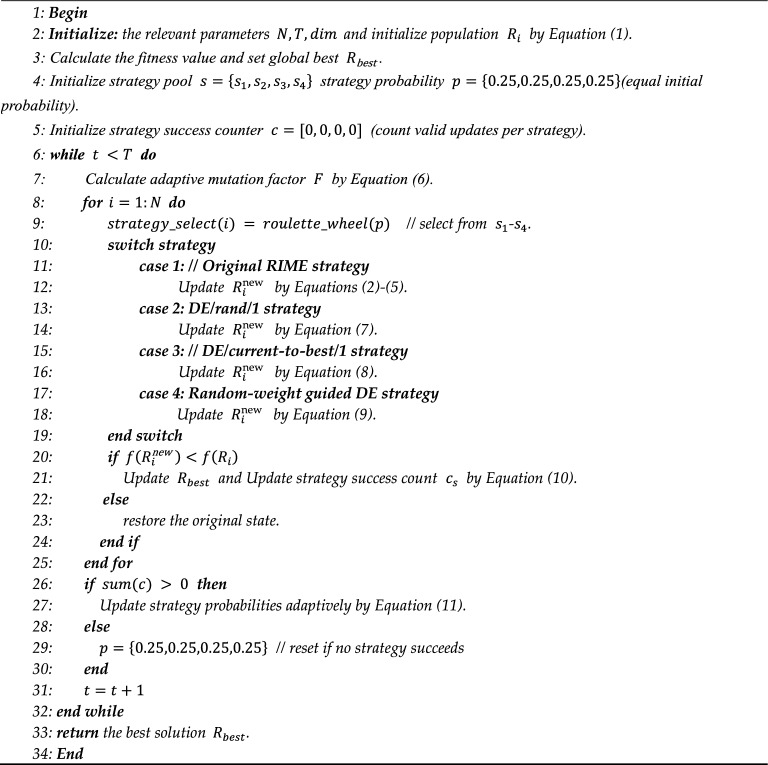
The pseudo code of the MSRIME

### Computational complexity analysis

The computational complexity of the proposed MSRIME algorithm is mainly determined by the population size $N$, problem dimension $D$, and the maximum number of iterations $T$. In each iteration, all individuals in the population are updated according to one of the strategies selected from the strategy pool. Each update operation involves vector-based computations with a cost proportional to the problem dimension, resulting in a per-iteration complexity of $O\left( {N \times D} \right)$. Therefore, the overall time complexity of MSRIME can be expressed as $O\left( {T \times N \times D} \right)$.

Compared with the standard RIME algorithm, MSRIME introduces three additional components: adaptive differential mutation factor adjustment, a multi-strategy collaborative update pool, and a probability-based strategy selection mechanism. However, these enhancements do not fundamentally change the computational complexity order. Specifically, the adaptive mutation factor is computed using a simple linear function with negligible cost; the strategy selection mechanism involves only basic probability calculations and roulette-wheel sampling with $O\left( 1 \right)$ complexity per individual; and although multiple update strategies are provided, only one strategy is applied to each individual per iteration. As a result, the computational overhead introduced by these improvements is limited to constant-level operations.

Therefore, MSRIME maintains the same asymptotic time complexity as the original RIME, i.e., $O\left( {T \times N \times D} \right)$, while achieving significant improvements in optimization performance. This demonstrates that the proposed enhancements improve efficiency without increasing algorithmic complexity.

## Numerical experiments

### Benchmarked algorithms and parameter configuration

In this subsection, the proposed MSRIME algorithm is evaluated using the currently most challenging numerical optimization benchmark, CEC2017^53^ and CEC2022^54^, and its performance is compared with several advanced optimization methods. The comparative algorithms include: Velocity Pausing Particle Swarm Optimization (VPPSO)^55^, Improved multi-strategy adaptive Grey Wolf Optimization (IAGWO)^11^, Improved Whale Optimization Algorithm (IWOA)^56^, Improving differential evolution through Bayesian hyperparameter optimization (MadDE)^57^, Status-based optimization Algorithm (SBO)^58^, Animated Oat Optimization (AOO)^59^, Holistic Swarm Optimization (HSO)^60^, and Rime optimization algorithm (RIME)^40^. he parameter settings for these algorithms are summarized in Table 2.

**Table 2:** Parameter settings of the compared algorithms.

Algorithms	Name of the parameter	Value of the parameter
VPPSO	$c_{1} ,c_{2} , w,\alpha ,N_{1} ,N_{2}$	2, 2, 0.8, [1,0], 0.15, 0.15
IAGWO	$v_{rand} \left( t \right),a, \omega ,\theta$	[− 20,20], [0,2], [0.3,0.9], 0.5
IWOA	$r,l,a$	[0,1], [−1,1], Linear reduction from 2 to 1
MadDE	$p,\left| A \right|,H$	$0.18 , 2.3, 10 \times dim$
SBO	$I,P_{S} ,{ }R$	$\left\{ {1,{ }2} \right\},{ }\left[ {0,{ }1} \right],{ }0.1$
AOO	$K,{ }\theta$	$\left[ {0.5,1.5\left] {,{ }} \right[0,\pi } \right]$
HSO	$\alpha$	3
RIME	$W$	5
MSRIME	$W,CR,p_{CR} ,F,p_{s}$	$5,{ }0.95,0.8, 0.25$

### Evaluation of ablation experiments

To evaluate the effectiveness of each proposed improvement on the optimization performance of the RIME algorithm, ablation experiments were designed. Using the CEC2017 benchmark function set (30 dimensions), the original RIME algorithm was compared with three single-strategy variants: RIME-S1 (adaptive differential mutation factor adjustment), RIME-S2 (multi-strategy collaborative update pool), and RIME-S3 (strategy probability adaptive selection mechanism), as well as the fully integrated MSRIME algorithm. The performance differences are summarized in Table 3 and illustrated in Figs. 1 and 3.

**Table 3:** Experimental outcomes for the CEC2017 benchmark (30-dimensional setting).

Function	Metric	RIME	RIME-S1	RIME-S2	RIME-S3	MSRIME
F1	Ave	3.6794E+06	8.3011E+05	2.0279E+05	1.6586E+04	**6.2878E+03**
	Std	1.4666E+06	6.9143E+05	1.5360E+05	5.4095E+04	**6.3125E+03**
F2	Ave	3.3853E+17	**2.7008E+16**	2.7771E+19	5.1086E+23	9.7698E+16
	Std	1.5986E+18	**1.0028E+17**	1.9620E+20	3.6123E+24	5.5307E+17
F3	Ave	4.9157E+04	4.3956E+04	**2.1253E+04**	7.5103E+04	2.7003E+04
	Std	1.6190E+04	1.4961E+04	**1.0625E+04**	2.3387E+04	1.7846E+04
F4	Ave	5.3232E+02	5.2061E+02	5.1022E+02	5.0378E+02	**4.9331E+02**
	Std	3.4941E+01	2.7375E+01	2.9341E+01	2.8819E+01	**2.1115E+01**
F5	Ave	6.1099E+02	6.1566E+02	6.1396E+02	6.0149E+02	**5.8317E+02**
	Std	2.8761E+01	2.8772E+01	2.8937E+01	3.0093E+01	**2.5285E+01**
F6	Ave	6.1537E+02	6.1219E+02	6.1334E+02	6.1143E+02	**6.0600E+02**
	Std	6.7517E+00	5.8766E+00	6.4136E+00	8.1890E+00	**5.1044E+00**
F7	Ave	8.7775E+02	8.5547E+02	8.5098E+02	8.4051E+02	**8.2521E+02**
	Std	4.0116E+01	3.9441E+01	3.3130E+01	4.0824E+01	**2.7795E+01**
F8	Ave	9.1387E+02	9.1300E+02	9.1794E+02	9.0257E+02	**8.8922E+02**
	Std	3.9948E+01	2.7787E+01	3.1449E+01	**2.6411E+01**	3.1185E+01
F9	Ave	3.2111E+03	2.9388E+03	3.3200E+03	2.2162E+03	**1.5643E+03**
	Std	1.7505E+03	1.5652E+03	1.7641E+03	**1.0973E+03**	1.3043E+03
F10	Ave	**4.6655E+03**	4.6956E+03	4.6875E+03	4.9259E+03	4.8326E+03
	Std	6.2445E+02	6.0652E+02	**5.9524E+02**	7.2790E+02	8.6801E+02
F11	Ave	1.3416E+03	1.3246E+03	1.3015E+03	**1.2915E+03**	1.2980E+03
	Std	5.7946E+01	**5.4975E+01**	8.2258E+01	6.2928E+01	7.3573E+01
F12	Ave	1.6088E+07	1.7559E+07	9.7147E+05	1.3522E+06	**5.0306E+05**
	Std	1.9826E+07	1.6301E+07	1.0749E+06	1.3918E+06	**9.0580E+05**
F13	Ave	1.5664E+05	6.6257E+04	3.0221E+04	2.3554E+04	**1.7948E+04**
	Std	2.5725E+05	1.1276E+05	2.5504E+04	2.3076E+04	**1.6908E+04**
F14	Ave	1.0141E+05	**1.5670E+03**	2.5849E+03	1.0580E+05	1.5929E+03
	Std	9.4891E+04	3.4684E+02	2.9648E+03	9.4938E+04	**1.1490E+02**
F15	Ave	1.9418E+04	1.4136E+04	1.3489E+04	**1.1489E+04**	1.6412E+04
	Std	**1.1517E+04**	1.2245E+04	1.5061E+04	1.2327E+04	1.4608E+04
F16	Ave	2.7352E+03	2.7478E+03	2.5883E+03	2.5654E+03	**2.4910E+03**
	Std	2.7472E+02	3.0898E+02	2.8814E+02	3.3854E+02	**2.7060E+02**
F17	Ave	2.2612E+03	2.2015E+03	2.2694E+03	2.1612E+03	**2.1498E+03**
	Std	2.4632E+02	**2.1077E+02**	2.3616E+02	2.1369E+02	2.3198E+02
F18	Ave	1.9150E+06	1.8264E+05	**9.2603E+04**	2.0032E+06	1.0677E+05
	Std	1.7701E+06	2.5917E+05	**7.1911E+04**	2.1202E+06	1.1463E+05
F19	Ave	1.7701E+04	1.4405E+04	1.7047E+04	**1.3826E+04**	1.5360E+04
	Std	**1.3527E+04**	1.5991E+04	1.8381E+04	1.4091E+04	1.6518E+04
F20	Ave	2.5390E+03	2.5125E+03	2.4935E+03	2.5206E+03	**2.4556E+03**
	Std	2.1386E+02	**2.0234E+02**	2.3066E+02	2.0503E+02	2.0319E+02
F21	Ave	2.4131E+03	2.4235E+03	2.4153E+03	2.3914E+03	**2.3819E+03**
	Std	2.6429E+01	3.0313E+01	2.6400E+01	2.6168E+01	**2.2683E+01**
F22	Ave	**4.7544E+03**	5.5674E+03	5.5982E+03	5.1215E+03	5.7921E+03
	Std	1.8920E+03	1.7401E+03	1.6708E+03	1.9592E+03	**1.5706E+03**
F23	Ave	2.7831E+03	2.7852E+03	2.7736E+03	2.7557E+03	**2.7442E+03**
	Std	3.5847E+01	**3.1582E+01**	4.0870E+01	3.9054E+01	3.4793E+01
F24	Ave	2.9530E+03	2.9535E+03	2.9409E+03	2.9265E+03	**2.9081E+03**
	Std	3.8082E+01	**3.2717E+01**	3.6915E+01	3.5873E+01	3.6941E+01
F25	Ave	2.9238E+03	2.9143E+03	2.9084E+03	2.8963E+03	**2.8910E+03**
	Std	2.7227E+01	2.5406E+01	2.0991E+01	1.4237E+01	**9.2104E+00**
F26	Ave	4.9723E+03	5.0669E+03	5.0544E+03	4.7908E+03	**4.6829E+03**
	Std	7.0280E+02	3.8242E+02	3.7545E+02	5.0180E+02	**3.5421E+02**
F27	Ave	3.2448E+03	3.2393E+03	3.2342E+03	3.2436E+03	**3.2268E+03**
	Std	**1.6382E+01**	2.0734E+01	2.2210E+01	2.3071E+01	1.8471E+01
F28	Ave	3.2868E+03	3.2864E+03	3.3308E+03	3.2520E+03	**3.2376E+03**
	Std	**3.1531E+01**	3.8539E+01	4.6546E+02	4.6788E+01	3.7223E+01
F29	Ave	4.0363E+03	3.9534E+03	3.9367E+03	3.9130E+03	**3.7857E+03**
	Std	**1.8013E+02**	2.2365E+02	2.4768E+02	2.0475E+02	2.0063E+02
F30	Ave	8.0296E+05	1.4844E+05	**1.4390E+04**	1.7201E+04	1.4516E+04
	Std	6.6215E+05	2.1597E+05	**5.8498E+03**	1.9004E+04	6.6464E+03

Significant values are in bold.

From Table 2 (CEC2017, 30-dimensional ablation experiment results), it can be seen that the proposed MSRIME achieves lower average fitness values (Ave) on most test functions compared to the original RIME and each single-strategy variant. In several functions, the improvement is particularly significant. For example, on F1, RIME’s average is 3.6794 × 10^6^, whereas MSRIME achieves 6.2878 × 10^3^, representing a reduction by several orders of magnitude; on F30, RIME’s average is 8.0296 × 10^5^, while MSRIME reaches 1.4516 × 10^4^, again showing clear improvement. Meanwhile, the standard deviation (Std) of MSRIME on these instances is also significantly smaller (e.g., F1’s Std decreases from 1.4666 × 10^6^ to 6.3125 × 10^3^), indicating that the algorithm not only performs better on average but is also more stable.

**Fig. 2:**
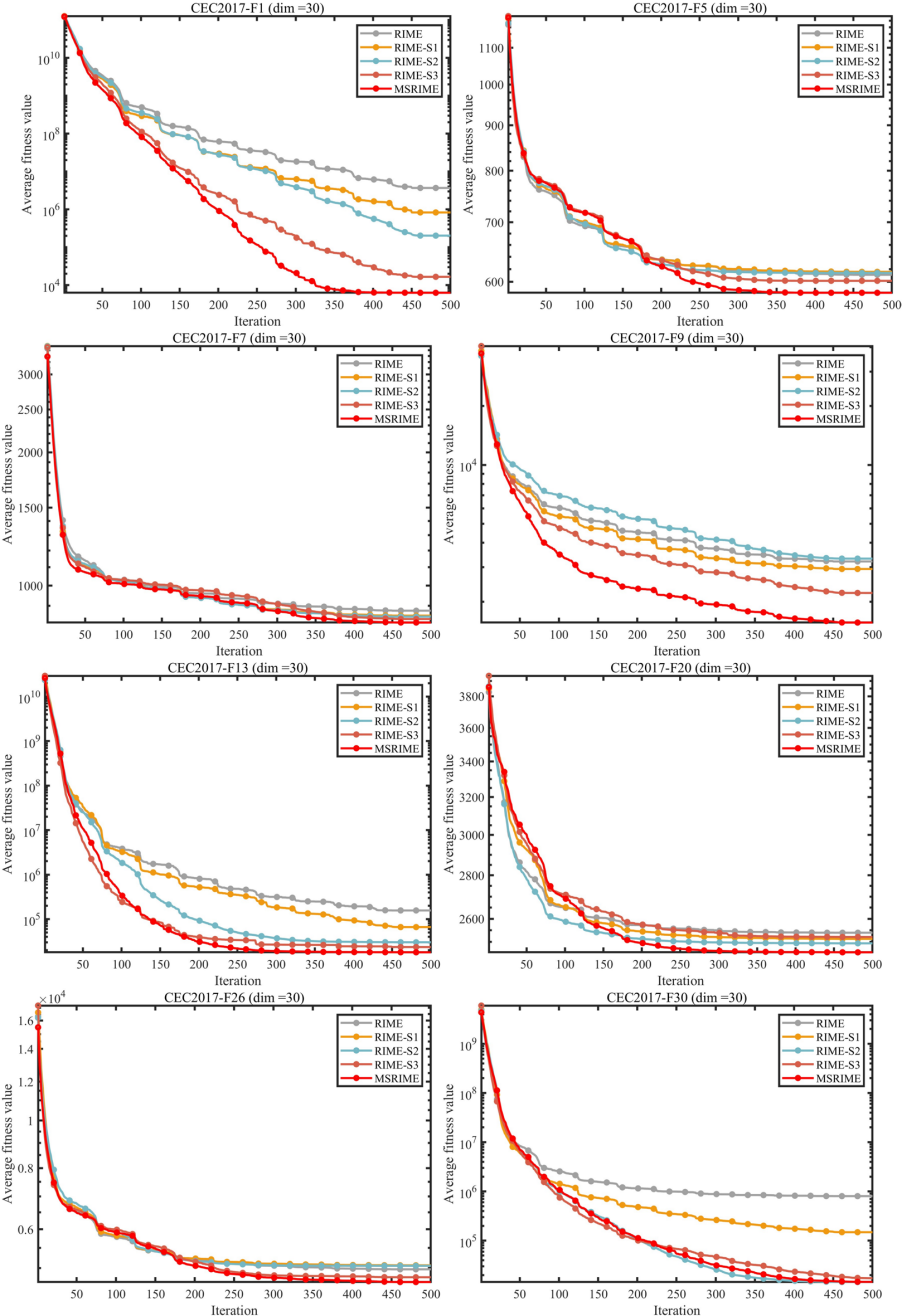
Convergence of enhanced RIME variants under various improvement strategies.

The convergence curves in Fig. 2 intuitively illustrate the optimization process. On functions such as F1, F5, and F7, MSRIME’s convergence curve consistently remains at the bottom and declines fastest, quickly approaching the optimal region within the first 50 iterations, whereas the original RIME and single-strategy variants show delayed convergence. Taking F9 as an example, MSRIME achieves stable convergence after 200 iterations, while RIME-S3 requires more than 300 iterations, and the original RIME curve remains at a high level. This indicates that the adaptive differential mutation factor enhances global exploration in the early stage, while the multi-strategy collaborative update and probability selection mechanisms accelerate mid-stage convergence and late-stage fine exploitation. Additionally, MSRIME exhibits smaller fluctuations throughout the iteration process; for F30, its convergence curve is significantly smoother than other algorithms, confirming the positive effect of multi-strategy integration on algorithm stability. This is consistent with the smaller mean and standard deviation observed in Table 2. The convergence trends in Fig. 2 thus provide a dynamic explanation of the numerical advantages shown in Table 2.

**Fig. 3:**
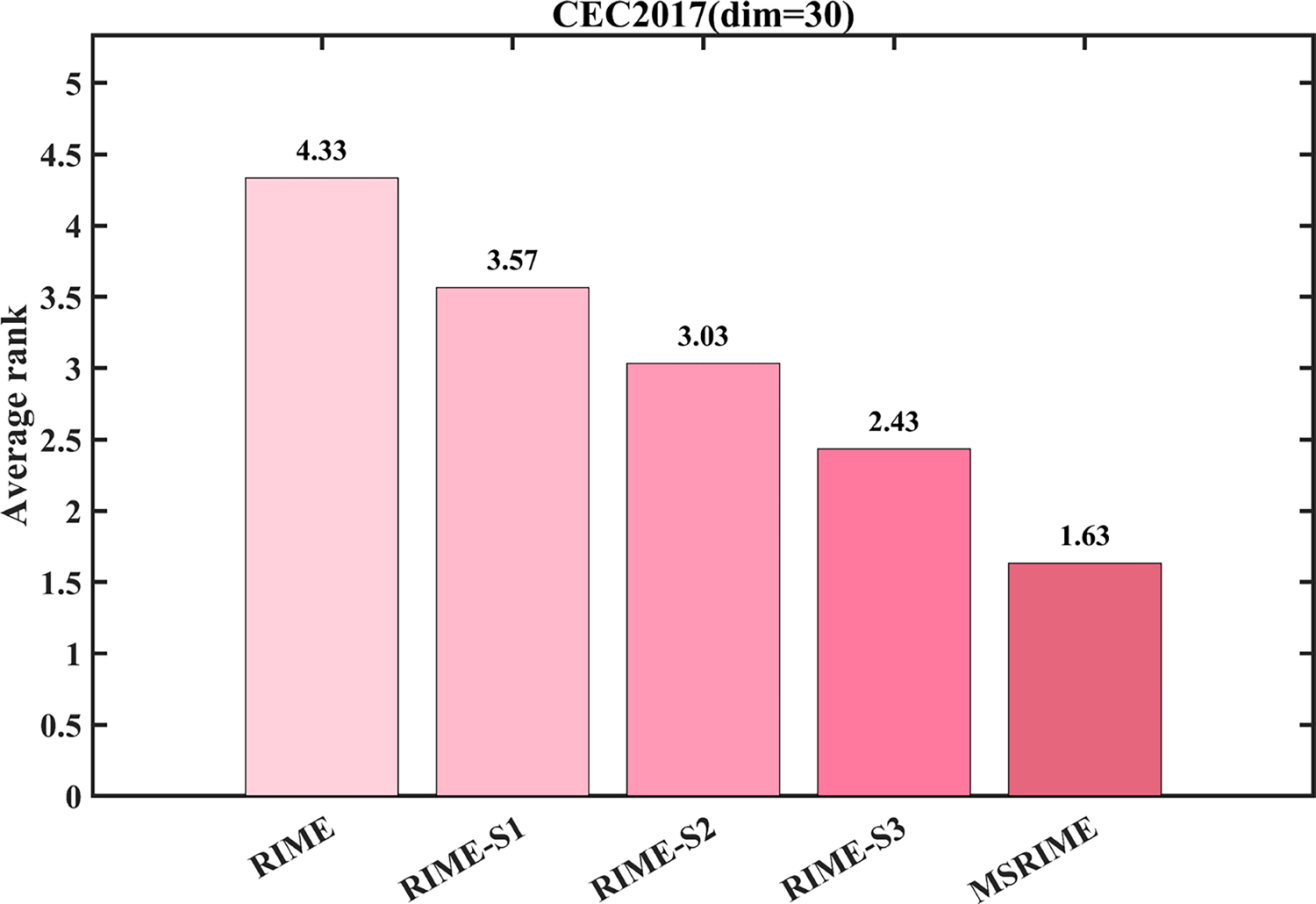
Mean ranking of enhanced RIME variants under various improvement strategies.

The average ranking results in Fig. 3 further quantify the overall performance of the algorithms. MSRIME achieves an average rank of 1.63, far superior to the original RIME (4.33) and other single-strategy variants (RIME-S1: 3.57, RIME-S2: 3.03, RIME-S3: 2.43), establishing it as the algorithm with the best comprehensive performance. This indicates that while single improvement strategies can enhance performance in specific cases (e.g., RIME-S3 ranks higher than RIME-S1 and RIME-S2 on some multimodal functions), they are limited in scope. MSRIME, through the deep integration of adaptive differential mutation factor adjustment, multi-strategy collaborative updates, and strategy probability adaptive selection, achieves a dynamic balance between exploration and exploitation, demonstrating superior robustness across different types of optimization problems.

In conclusion, the numerical values in Table 2, the convergence curves in Fig. 2, and the average rankings in Fig. 3 form three mutually supporting lines of evidence: MSRIME not only attains better final solution quality (lower objective values) on most CEC2017 test functions, but also converges faster, runs more stably (smaller standard deviations), and ranks highest in overall statistical evaluation. These results confirm that the multi-strategy adaptive design is effective in balancing exploration and exploitation while improving robustness.

### Evaluation on the CEC2017 and CEC2022Benchmark

**Fig. 4:**
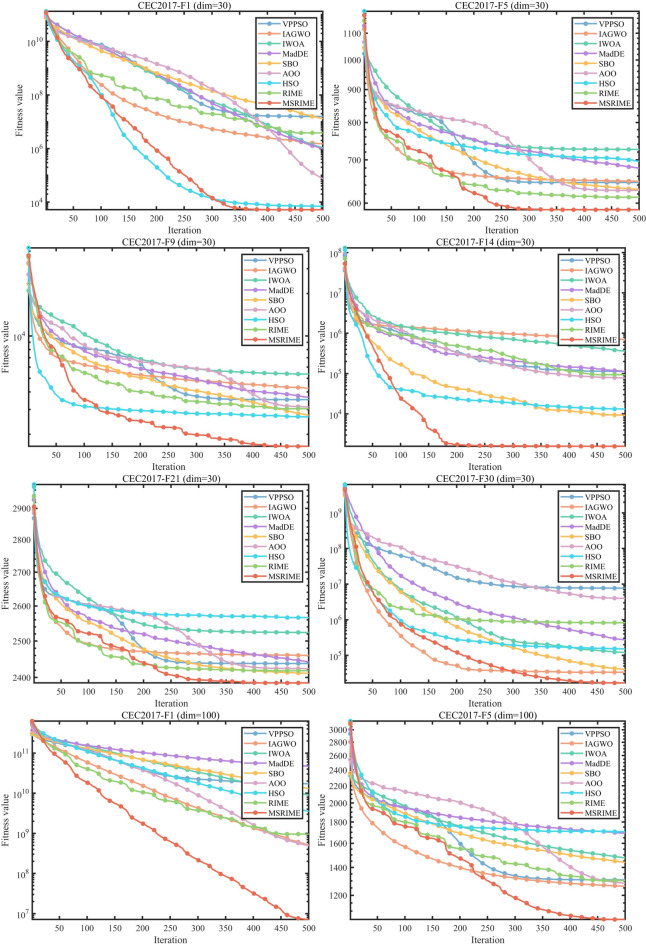

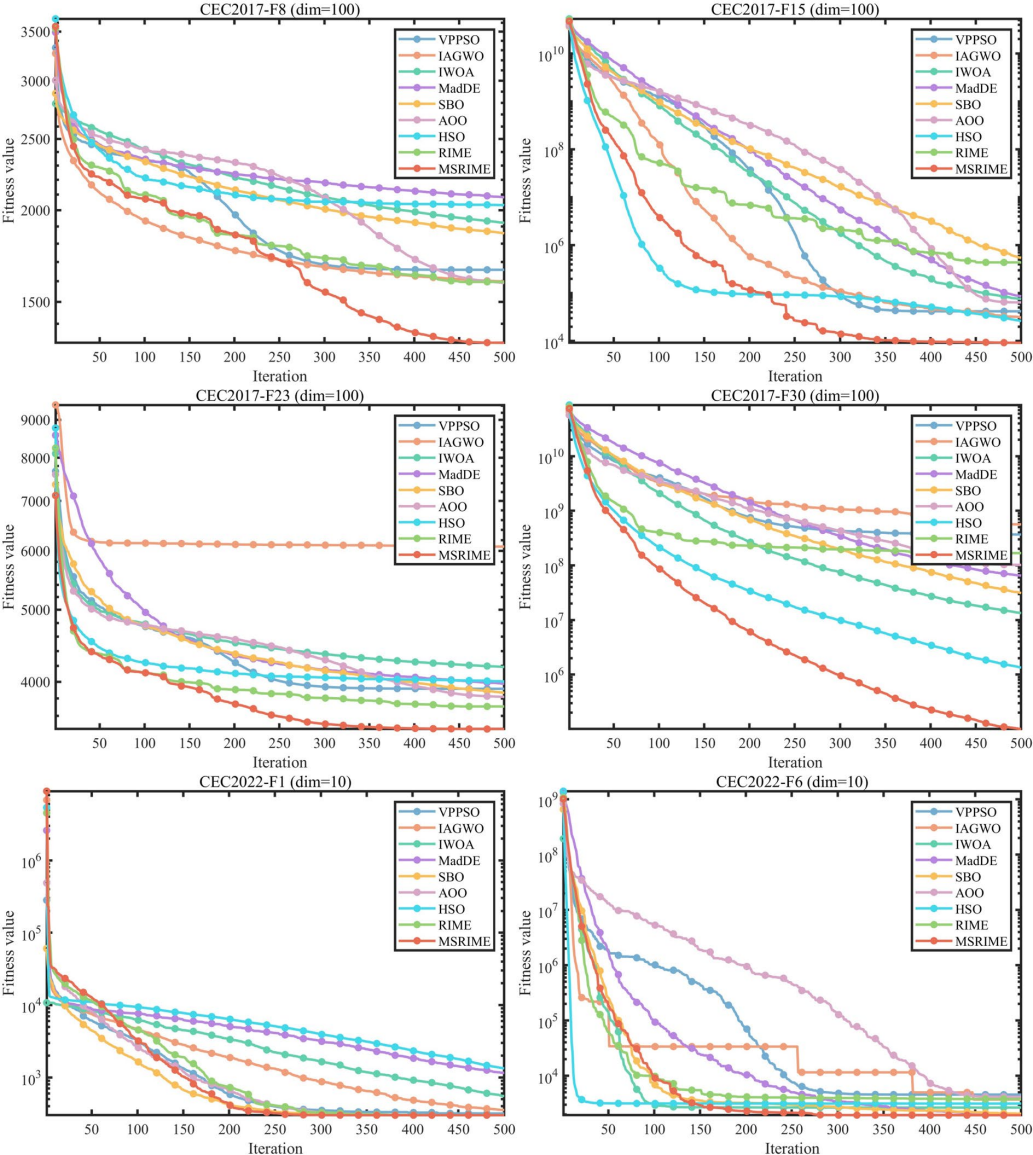

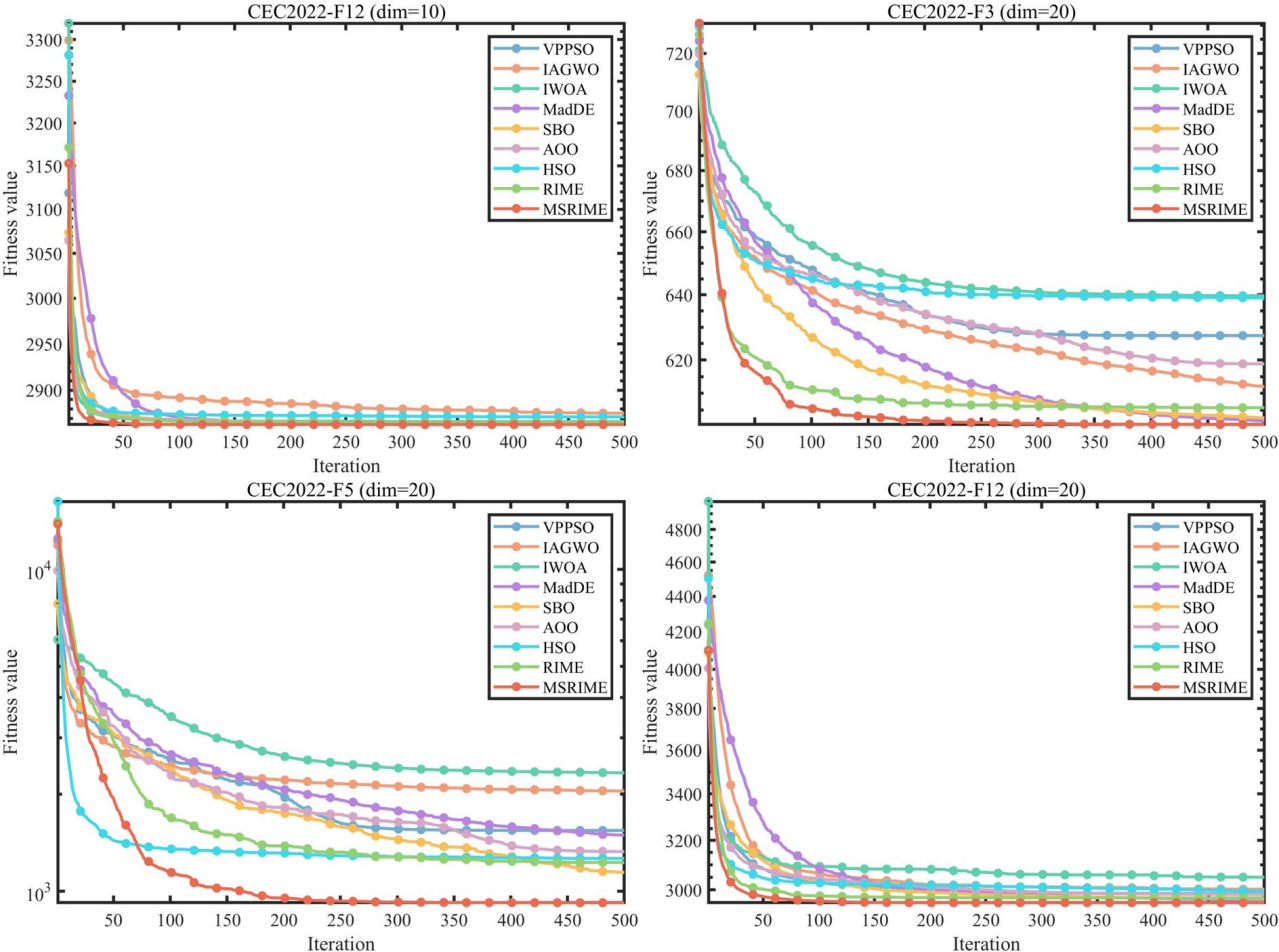
Convergence rates of various algorithms evaluated on the benchmark test set.

To guarantee impartial evaluation and minimize chance effects, every method adopts consistent settings: population size at thirty, maximum generations fixed at five hundred. Each technique executes independently thirty times. Outcome data report average values, standard deviation, plus relative standings, with best performances bolded. Computations occur under this uniform platform: Microsoft Windows 10, thirteenth-generation Intel Core i5-13400 CPU operating at 2.5 gigahertz, sixteen gigabytes RAM, utilizing MATLAB 2024b. This standardized framework and analytical approach confirm findings are dependable and permit direct comparisons. The results are displayed in Tables 4, 5, 6, 7 and Fig. 4.

**Table 4:** Experimental Outcomes for the CEC2017 Benchmark (30-Dimensional Setting).

Function	Metric	VPPSO	IAGWO	IWOA	MadDE	SBO	AOO	HSO	RIME	MSRIME
F1	Ave	1.5805E+07	1.5144E+06	1.0590E+06	9.7873E+05	1.2977E+07	7.3666E+04	6.9748E+03	3.8513E+06	**5.1720E+03**
	Std	4.2689E+07	5.9319E+05	7.9189E+05	6.7655E+05	8.2689E+06	3.3644E+04	6.6418E+03	1.3492E+06	**5.8439E+03**
F2	Ave	2.1486E+23	3.1031E+34	7.7841E+20	1.4001E+21	1.5315E+20	3.7572E+16	**9.5391E+14**	7.7743E+17	3.6624E+18
	Std	1.0735E+24	1.5327E+35	2.5824E+21	7.3048E+21	7.8736E+20	8.0076E+16	**2.0256E+15**	2.0324E+18	2.0040E+19
F3	Ave	4.7294E+04	6.3927E+04	1.6085E+05	7.8918E+04	**1.7926E+04**	2.6511E+04	4.9068E+04	4.7834E+04	1.9823E+04
	Std	1.4172E+04	2.6042E+04	3.0164E+04	1.0847E+04	**6.1593E+03**	1.0045E+04	1.2408E+04	1.5351E+04	9.0147E+03
F4	Ave	5.2549E+02	5.8175E+02	5.2121E+02	5.1479E+02	5.3915E+02	5.0493E+02	6.9649E+02	5.2670E+02	**5.0091E+02**
	Std	3.0792E+01	7.2009E+01	2.4718E+01	**1.3137E+01**	3.6301E+01	2.0591E+01	1.0950E+02	3.6875E+01	2.2456E+01
F5	Ave	6.4587E+02	6.4848E+02	7.2672E+02	6.7973E+02	6.3031E+02	6.2775E+02	6.9656E+02	6.1285E+02	**5.8596E+02**
	Std	3.2567E+01	2.8854E+01	5.3120E+01	**1.9631E+01**	3.5755E+01	2.9918E+01	2.7380E+01	3.0762E+01	2.2148E+01
F6	Ave	6.3529E+02	6.2069E+02	6.5676E+02	6.1009E+02	6.1215E+02	6.2985E+02	6.5039E+02	6.1435E+02	**6.0491E+02**
	Std	9.2130E+00	9.4554E+00	1.1082E+01	**3.7695E+00**	5.2825E+00	8.7904E+00	4.2055E+00	4.8518E+00	4.2711E+00
F7	Ave	9.3896E+02	9.3103E+02	1.0701E+03	9.7202E+02	9.2017E+02	8.5688E+02	1.0621E+03	8.7898E+02	**8.1928E+02**
	Std	7.3200E+01	4.1436E+01	9.8985E+01	3.1294E+01	3.9362E+01	2.9535E+01	7.8777E+01	3.6035E+01	**2.7025E+01**
F8	Ave	9.2742E+02	9.1872E+02	9.8460E+02	9.4308E+02	9.2551E+02	9.0347E+02	1.0541E+03	9.1701E+02	**8.9424E+02**
	Std	3.5795E+01	2.1189E+01	3.0264E+01	**1.7847E+01**	2.8255E+01	2.7551E+01	2.4121E+01	2.9073E+01	3.4607E+01
F9	Ave	3.5252E+03	4.2456E+03	5.3487E+03	3.6542E+03	2.7355E+03	3.1268E+03	2.6542E+03	3.0389E+03	**1.6409E+03**
	Std	1.0757E+03	9.5032E+02	8.8939E+02	8.8485E+02	1.0611E+03	1.3572E+03	**7.0895E+02**	1.5840E+03	8.1963E+02
F10	Ave	5.0476E+03	4.4045E+03	5.5908E+03	5.8028E+03	5.5901E+03	4.6802E+03	**4.2278E+03**	4.7301E+03	5.0237E+03
	Std	7.9732E+02	5.5063E+02	6.9796E+02	**3.6592E+02**	1.4282E+03	6.8441E+02	4.8370E+02	6.0371E+02	1.0164E+03
F11	Ave	1.4057E+03	1.9952E+03	1.3451E+03	1.3232E+03	**1.2379E+03**	1.2836E+03	1.7859E+03	1.3340E+03	1.2909E+03
	Std	1.3843E+02	1.1544E+03	8.4985E+01	4.2581E+01	**3.1968E+01**	4.9189E+01	2.1118E+02	7.5905E+01	6.8718E+01
F12	Ave	2.1627E+07	2.9081E+07	4.8159E+06	2.2796E+06	3.3350E+06	1.4803E+07	**4.5966E+05**	2.3242E+07	6.1960E+05
	Std	1.6263E+07	4.5632E+07	4.6357E+06	1.0471E+06	3.2837E+06	1.3539E+07	6.9564E+05	2.1360E+07	**5.1114E+05**
F13	Ave	1.0817E+05	2.8943E+05	4.2798E+04	8.3862E+04	2.6710E+04	9.8542E+04	**2.0895E+04**	1.9978E+05	2.2177E+04
	Std	5.7138E+04	1.4680E+06	3.6053E+04	7.5954E+04	2.4168E+04	6.7188E+04	**1.2875E+04**	2.1870E+05	2.1287E+04
F14	Ave	1.1350E+05	6.9842E+05	3.6259E+05	1.1320E+05	9.2753E+03	7.7723E+04	1.3233E+04	9.3913E+04	**1.5684E+03**
	Std	7.9945E+04	5.9568E+05	4.3501E+05	1.0440E+05	8.1090E+03	5.2881E+04	1.0624E+04	7.6440E+04	**7.9737E+01**
F15	Ave	8.3961E+04	**4.5474E+03**	1.4695E+04	8.2081E+03	9.0974E+03	6.3079E+04	9.1173E+03	2.3705E+04	1.1378E+04
	Std	2.2203E+05	**3.3501E+03**	1.4244E+04	4.3995E+03	8.5210E+03	4.1101E+04	4.2835E+03	1.7931E+04	1.2395E+04
F16	Ave	2.9454E+03	2.9660E+03	2.9070E+03	2.6410E+03	2.6582E+03	2.6744E+03	2.9087E+03	2.6578E+03	**2.4818E+03**
	Std	3.1767E+02	3.8421E+02	3.2089E+02	**1.5334E+02**	2.8037E+02	2.7155E+02	2.6152E+02	3.7502E+02	3.0976E+02
F17	Ave	2.2056E+03	2.3987E+03	2.5793E+03	**2.0049E+03**	2.0926E+03	2.1719E+03	2.6294E+03	2.2101E+03	2.0944E+03
	Std	1.9520E+02	3.1477E+02	2.6185E+02	**1.0965E+02**	2.4126E+02	1.7968E+02	2.7266E+02	2.2459E+02	1.9087E+02
F18	Ave	1.1948E+06	1.8710E+06	2.5649E+06	5.4386E+05	1.9385E+05	1.0246E+06	1.8146E+05	1.3500E+06	**8.2352E+04**
	Std	1.1162E+06	3.3168E+06	2.2042E+06	3.3249E+05	2.1161E+05	1.0456E+06	1.6502E+05	9.9128E+05	**8.1946E+04**
F19	Ave	1.9128E+06	7.6385E+03	1.5028E+04	9.2620E+03	**5.4890E+03**	6.5460E+05	2.7888E+04	1.6686E+04	1.7870E+04
	Std	1.1924E+06	6.9289E+03	2.6346E+04	5.5177E+03	**3.9704E+03**	6.6186E+05	6.0721E+04	1.3223E+04	2.0175E+04
F20	Ave	2.5202E+03	2.6402E+03	2.7387E+03	2.3909E+03	**2.3787E+03**	2.5449E+03	2.4898E+03	2.5357E+03	2.4795E+03
	Std	1.6934E+02	1.9209E+02	1.8670E+02	**1.1579E+02**	1.3114E+02	2.0356E+02	1.9062E+02	2.0412E+02	2.1190E+02
F21	Ave	2.4380E+03	2.4600E+03	2.5239E+03	2.4425E+03	2.4115E+03	2.4242E+03	2.5665E+03	2.4183E+03	**2.3854E+03**
	Std	3.6698E+01	2.6297E+01	5.4386E+01	3.5218E+01	2.6931E+01	4.0877E+01	**1.7136E+01**	2.9754E+01	2.8184E+01
F22	Ave	3.6418E+03	3.7250E+03	3.5188E+03	2.5222E+03	**2.3229E+03**	4.8376E+03	4.5796E+03	4.8762E+03	5.4765E+03
	Std	2.0498E+03	2.0362E+03	2.2471E+03	1.0474E+03	**4.6395E+00**	2.0808E+03	1.6299E+03	1.9763E+03	1.5681E+03
F23	Ave	2.8053E+03	3.0714E+03	2.8968E+03	2.8220E+03	2.7690E+03	2.7985E+03	2.9099E+03	2.7953E+03	**2.7392E+03**
	Std	4.8878E+01	1.5375E+02	8.2308E+01	2.1029E+01	3.1331E+01	5.5709E+01	**1.5880E+01**	4.5958E+01	2.6211E+01
F24	Ave	2.9544E+03	3.3039E+03	3.0126E+03	2.9992E+03	2.9599E+03	2.9624E+03	3.0443E+03	2.9476E+03	**2.9100E+03**
	Std	2.9956E+01	1.5789E+02	5.1092E+01	2.4274E+01	3.6860E+01	3.9343E+01	**1.4911E+01**	3.7662E+01	3.2368E+01
F25	Ave	2.9485E+03	2.9546E+03	2.9142E+03	2.9253E+03	2.9275E+03	2.9079E+03	3.1775E+03	2.9225E+03	**2.8906E+03**
	Std	2.5331E+01	3.0770E+01	2.3220E+01	1.8742E+01	1.8946E+01	2.1945E+01	9.0941E+01	3.1613E+01	**1.0691E+01**
F26	Ave	4.9214E+03	5.3448E+03	5.6086E+03	**3.5347E+03**	4.2865E+03	4.7674E+03	5.3571E+03	5.0408E+03	4.6084E+03
	Std	1.3199E+03	1.8621E+03	1.4328E+03	1.1004E+03	1.2925E+03	1.0078E+03	**3.7127E+02**	5.8565E+02	4.5754E+02
F27	Ave	3.3032E+03	**3.2249E+03**	3.4564E+03	3.2520E+03	3.2641E+03	3.2558E+03	3.3249E+03	3.2444E+03	3.2282E+03
	Std	6.1182E+01	8.3277E+01	1.3210E+02	**8.9937E+00**	2.7067E+01	1.9760E+01	4.6553E+01	1.4888E+01	1.8782E+01
F28	Ave	3.3218E+03	3.4070E+03	4.2829E+03	3.2820E+03	3.2794E+03	3.2530E+03	3.6806E+03	3.2998E+03	**3.2266E+03**
	Std	3.7980E+01	1.7034E+02	1.1383E+03	2.1614E+01	2.7576E+01	2.8739E+01	2.9430E+02	4.2628E+01	**2.0999E+01**
F29	Ave	4.3415E+03	4.0313E+03	4.2513E+03	3.9054E+03	**3.8059E+03**	4.0236E+03	4.4601E+03	4.0775E+03	3.8146E+03
	Std	3.0416E+02	3.2398E+02	2.9103E+02	**1.2793E+02**	2.3704E+02	2.4276E+02	2.6561E+02	2.0900E+02	2.7833E+02
F30	Ave	7.7201E+06	3.3518E+04	1.1766E+05	2.7000E+05	4.0485E+04	3.9622E+06	1.5177E+05	8.2300E+05	**1.6791E+04**
	Std	4.4645E+06	1.5453E+05	1.5752E+05	1.3758E+05	3.0291E+04	2.0940E+06	1.8842E+05	5.2111E+05	**7.9783E+03**

Significant values are in bold.

**Table 5:** Experimental outcomes for the CEC2017 benchmark (100-dimensional setting).

Function	Metric	VPPSO	IAGWO	IWOA	MadDE	SBO	AOO	HSO	RIME	MSRIME
F1	Ave	1.7020E+10	5.1295E+08	8.9450E+09	4.7135E+10	1.3066E+10	4.8888E+08	3.4964E+09	9.5576E+08	**7.1034E+06**
	Std	4.0782E+09	7.5116E+08	2.1810E+09	8.2033E+09	2.4491E+09	1.6033E+08	1.8222E+09	2.0505E+08	**7.6237E+06**
F2	Ave	4.2658E+141	5.7107E+155	3.2027E+136	**1.0000E+30**	1.0143E+128	1.4681E+116	3.2558E+201	3.7362E+129	1.8952E+112
	Std	2.3365E+142	**6.5535E+04**	1.6136E+137	1.4314E+14	3.6279E+128	3.8778E+116	**6.5535E+04**	2.0464E+130	1.0378E+113
F3	Ave	3.8718E+05	4.4536E+05	8.9390E+05	4.6012E+05	**2.8358E+05**	5.6769E+05	3.7406E+05	7.2619E+05	7.0902E+05
	Std	3.7288E+04	9.7643E+04	1.4126E+05	5.2279E+04	**2.4254E+04**	8.7225E+04	4.1258E+04	1.0586E+05	1.3506E+05
F4	Ave	2.9741E+03	2.7967E+03	1.9016E+03	4.4818E+03	2.9981E+03	1.0810E+03	2.9985E+03	1.2280E+03	**8.3992E+02**
	Std	5.1726E+02	2.9327E+03	2.9456E+02	8.7227E+02	7.4401E+02	9.3296E+01	7.9310E+02	1.1944E+02	**7.6169E+01**
F5	Ave	1.3077E+03	1.2621E+03	1.4767E+03	1.6919E+03	1.4408E+03	1.2819E+03	1.7062E+03	1.3022E+03	**1.0495E+03**
	Std	8.4608E+01	6.6208E+01	6.7843E+01	**3.9563E+01**	6.0097E+01	1.1508E+02	7.5738E+01	9.6619E+01	8.5754E+01
F6	Ave	6.6261E+02	6.4705E+02	6.7410E+02	6.7007E+02	6.6506E+02	6.6461E+02	6.9074E+02	6.5933E+02	**6.4459E+02**
	Std	4.7035E+00	5.7848E+00	**3.1729E+00**	4.0487E+00	6.0614E+00	5.0611E+00	4.4579E+00	6.3331E+00	7.1936E+00
F7	Ave	2.5292E+03	2.2207E+03	3.1253E+03	2.9867E+03	2.7322E+03	2.0203E+03	4.7176E+03	2.2287E+03	**1.6803E+03**
	Std	3.2972E+02	**1.5185E+02**	2.0911E+02	1.9737E+02	2.2913E+02	1.9074E+02	5.8210E+02	2.6864E+02	1.5610E+02
F8	Ave	1.6585E+03	1.5985E+03	1.9215E+03	2.0823E+03	1.8598E+03	1.5912E+03	2.0305E+03	1.5969E+03	**1.3190E+03**
	Std	1.0040E+02	9.4680E+01	6.4209E+01	**4.0451E+01**	8.4056E+01	1.1032E+02	6.9792E+01	1.1867E+02	9.7617E+01
F9	Ave	**2.5307E+04**	3.7967E+04	3.5384E+04	5.7713E+04	5.3060E+04	3.8232E+04	6.4892E+04	5.3785E+04	2.8873E+04
	Std	**2.5951E+03**	6.0977E+03	3.7215E+03	4.6328E+03	1.1293E+04	8.7414E+03	2.0020E+04	1.5773E+04	1.2734E+04
F10	Ave	1.8186E+04	**1.7068E+04**	1.9562E+04	2.7246E+04	2.1098E+04	1.7686E+04	2.3384E+04	1.9625E+04	1.8390E+04
	Std	3.0658E+03	3.6007E+03	1.6063E+03	**8.0022E+02**	3.2914E+03	1.2701E+03	1.9244E+03	1.3230E+03	1.9857E+03
F11	Ave	7.4004E+04	8.3708E+04	1.4495E+05	1.0334E+05	4.5314E+04	3.2318E+04	6.8758E+04	4.5954E+04	**3.2154E+04**
	Std	1.6868E+04	3.2694E+04	3.6828E+04	1.3873E+04	1.1168E+04	**8.1665E+03**	2.0700E+04	1.5058E+04	1.3695E+04
F12	Ave	1.1643E+09	7.8099E+09	7.7905E+08	3.2380E+09	1.7758E+09	5.5582E+08	1.9810E+08	1.0648E+09	**6.8801E+07**
	Std	4.3087E+08	1.5484E+10	2.3131E+08	1.4113E+09	8.1477E+08	1.4553E+08	1.3365E+08	3.4618E+08	**4.1893E+07**
F13	Ave	2.4746E+06	2.3188E+05	1.0773E+06	1.8941E+07	1.9287E+07	7.6866E+04	6.7703E+04	2.2309E+06	**1.0707E+04**
	Std	1.3174E+07	4.7099E+05	7.1408E+05	2.1945E+07	1.4516E+07	1.6638E+04	2.3613E+04	1.8423E+06	**5.0643E+03**
F14	Ave	6.3595E+06	1.3952E+07	4.6109E+06	7.6828E+06	2.3322E+06	4.2010E+06	**1.0321E+06**	7.6372E+06	1.2500E+06
	Std	3.1135E+06	1.7912E+07	1.5158E+06	2.1862E+06	1.2819E+06	1.7849E+06	9.0683E+05	3.7763E+06	**7.6129E+05**
F15	Ave	4.1756E+04	3.0842E+04	7.5105E+04	8.5530E+04	5.4645E+05	6.4074E+04	2.6347E+04	4.3806E+05	**9.1753E+03**
	Std	1.4687E+04	7.3086E+04	2.9566E+04	8.0014E+04	4.8477E+05	3.4644E+04	**6.5755E+03**	2.3362E+05	9.8461E+03
F16	Ave	7.9035E+03	6.9863E+03	7.0759E+03	9.6395E+03	6.6791E+03	6.5783E+03	7.1379E+03	7.6963E+03	**5.7893E+03**
	Std	8.4955E+02	1.5476E+03	9.5819E+02	**5.9797E+02**	7.4907E+02	7.1709E+02	7.2572E+02	8.2772E+02	6.6349E+02
F17	Ave	5.5095E+03	2.7824E+04	6.2969E+03	6.1570E+03	5.5530E+03	**5.3881E+03**	5.6522E+03	5.9087E+03	5.4680E+03
	Std	6.0833E+02	7.7705E+04	7.0006E+02	4.7755E+02	6.5342E+02	**4.6185E+02**	4.7339E+02	5.3071E+02	6.0456E+02
F18	Ave	4.3824E+06	1.1399E+07	6.3234E+06	6.9361E+06	3.0286E+06	5.2642E+06	**1.9382E+06**	1.1524E+07	3.8156E+06
	Std	2.3852E+06	3.2064E+07	3.1030E+06	2.1719E+06	1.3459E+06	3.0540E+06	**1.1228E+06**	5.7047E+06	2.9396E+06
F19	Ave	9.6188E+06	1.5667E+04	6.6194E+05	2.6386E+05	9.2806E+05	6.1511E+06	2.3851E+04	1.8886E+07	**7.8207E+03**
	Std	6.9842E+06	5.9005E+04	7.1591E+05	1.6566E+05	8.9734E+05	4.8309E+06	3.0173E+04	1.1630E+07	**7.8087E+03**
F20	Ave	5.3374E+03	5.5517E+03	5.9146E+03	6.2711E+03	5.3597E+03	5.3602E+03	**5.1248E+03**	5.8112E+03	5.6966E+03
	Std	5.2291E+02	4.1186E+02	7.6189E+02	**3.4282E+02**	5.2746E+02	5.6391E+02	5.1240E+02	5.4723E+02	8.1714E+02
F21	Ave	3.2041E+03	4.0368E+03	3.5854E+03	3.4980E+03	3.3592E+03	3.1352E+03	3.7111E+03	3.1769E+03	**2.9392E+03**
	Std	1.3677E+02	6.3795E+02	1.6935E+02	6.3983E+01	9.9589E+01	1.2909E+02	**5.9350E+01**	1.2359E+02	1.1891E+02
F22	Ave	2.0848E+04	2.0595E+04	2.3016E+04	3.0082E+04	2.4431E+04	**2.0060E+04**	2.5851E+04	2.1928E+04	2.1628E+04
	Std	1.5647E+03	3.8628E+03	1.1396E+03	**7.1063E+02**	3.2365E+03	1.9556E+03	1.7997E+03	1.7335E+03	4.9169E+03
F23	Ave	3.9145E+03	6.0793E+03	4.1894E+03	3.9759E+03	3.8655E+03	3.8178E+03	4.0044E+03	3.7062E+03	**3.4559E+03**
	Std	1.4202E+02	4.7014E+02	2.4332E+02	6.6194E+01	1.1941E+02	1.3337E+02	**4.8312E+01**	1.1560E+02	1.4046E+02
F24	Ave	4.6423E+03	7.1960E+03	5.0897E+03	4.6602E+03	4.7063E+03	4.5201E+03	4.5985E+03	4.2579E+03	**3.9095E+03**
	Std	2.0225E+02	8.7181E+02	3.2605E+02	8.9865E+01	1.7247E+02	1.8599E+02	**8.5852E+01**	1.1915E+02	1.2512E+02
F25	Ave	5.1360E+03	4.8012E+03	4.3921E+03	6.5049E+03	5.0382E+03	3.8425E+03	6.3242E+03	3.9336E+03	**3.5623E+03**
	Std	3.7562E+02	8.5733E+02	2.7021E+02	7.5263E+02	3.6284E+02	1.1155E+02	7.7453E+02	1.2622E+02	**1.0106E+02**
F26	Ave	2.2858E+04	1.9784E+04	2.2365E+04	2.5415E+04	1.9040E+04	1.6694E+04	1.9235E+04	1.5930E+04	**1.2195E+04**
	Std	2.9382E+03	6.1556E+03	5.3835E+03	2.2005E+03	6.5251E+03	3.2420E+03	2.0662E+03	1.8251E+03	**1.2748E+03**
F27	Ave	4.4469E+03	6.0294E+03	6.8121E+03	4.3769E+03	4.4091E+03	4.0087E+03	4.2879E+03	4.0233E+03	**3.6617E+03**
	Std	2.3605E+02	2.9385E+03	1.6702E+03	1.7201E+02	2.2639E+02	1.4815E+02	2.7740E+02	**1.1937E+02**	1.3463E+02
F28	Ave	6.0152E+03	6.8036E+03	1.8041E+04	8.9071E+03	6.3766E+03	3.9517E+03	1.4654E+04	4.2039E+03	**3.6835E+03**
	Std	6.3567E+02	3.8207E+03	7.5611E+03	9.6733E+02	6.3746E+02	**1.4666E+02**	5.2273E+03	2.9998E+02	1.7511E+02
F29	Ave	1.0180E+04	8.3095E+03	8.3329E+03	1.0669E+04	8.6758E+03	9.0107E+03	9.6669E+03	9.7145E+03	**7.4515E+03**
	Std	7.6561E+02	2.9105E+03	6.4316E+02	**5.6069E+02**	6.5448E+02	7.4599E+02	7.3506E+02	8.5092E+02	1.0760E+03
F30	Ave	3.6791E+08	5.6461E+08	1.3358E+07	6.4691E+07	3.1197E+07	1.0176E+08	1.3414E+06	1.6770E+08	**1.0039E+05**
	Std	1.9886E+08	2.4536E+09	1.1223E+07	3.6746E+07	1.6023E+07	3.6646E+07	8.6277E+05	8.6370E+07	**7.1189E+04**

Significant values are in bold.

**Table 6:** Experimental outcomes for the CEC2022 benchmark (10-dimensional setting).

Function	Metric	VPPSO	IAGWO	IWOA	MadDE	SBO	AOO	HSO	RIME	MSRIME
F1	Ave	3.1696E+02	3.5437E+02	5.5156E+02	1.1571E+03	3.0009E+02	3.0001E+02	1.3340E+03	3.0068E+02	**3.0000E+02**
	Std	3.8299E+01	6.9780E+01	2.3569E+02	7.2890E+02	2.1049E−01	5.9014E−03	2.6379E+02	6.2663E−01	**1.9269E−06**
F2	Ave	4.1220E+02	4.3650E+02	4.1272E+02	**4.0066E+02**	4.0436E+02	4.0630E+02	4.4130E+02	4.1318E+02	4.0629E+02
	Std	1.9578E+01	3.3933E+01	2.0040E+01	**1.9014E+00**	4.2943E+00	3.4004E+00	2.7785E+01	2.4208E+01	3.3164E+00
F3	Ave	6.0669E+02	6.0028E+02	6.1335E+02	6.0003E+02	6.0004E+02	6.0358E+02	6.1842E+02	6.0024E+02	**6.0001E+02**
	Std	5.6101E+00	1.2790E−01	1.2900E+01	**1.8511E−02**	3.8494E−02	4.4322E+00	4.9187E+00	1.4780E−01	3.4683E−02
F4	Ave	8.1837E+02	8.2087E+02	8.3379E+02	**8.1315E+02**	8.2084E+02	8.1961E+02	8.3994E+02	8.2232E+02	8.1755E+02
	Std	6.7720E+00	6.9593E+00	1.2266E+01	**2.4718E+00**	7.9357E+00	7.3115E+00	5.8295E+00	1.0923E+01	8.5302E+00
F5	Ave	9.1476E+02	9.3771E+02	1.0999E+03	9.0320E+02	9.0370E+02	9.0176E+02	9.0890E+02	9.0074E+02	**9.0007E+02**
	Std	2.1212E+01	9.6370E+01	1.8967E+02	4.9839E+00	9.1864E+00	6.0975E+00	9.0508E+00	1.5179E+00	**1.7294E−01**
F6	Ave	4.5589E+03	3.7109E+03	2.6579E+03	2.0165E+03	2.0625E+03	4.1630E+03	3.1391E+03	3.7986E+03	**1.9338E+03**
	Std	2.2794E+03	6.0828E+03	1.3901E+03	**2.4998E+02**	4.9585E+02	1.9207E+03	1.3706E+03	2.1378E+03	6.0907E+02
F7	Ave	2.0402E+03	2.0164E+03	2.0359E+03	**2.0069E+03**	2.0088E+03	2.0354E+03	2.0653E+03	2.0167E+03	2.0180E+03
	Std	1.1614E+01	8.6946E+00	1.5465E+01	**5.6937E+00**	9.0331E+00	1.3085E+01	2.4676E+01	8.0675E+00	6.9077E+00
F8	Ave	2.2228E+03	2.2208E+03	2.2265E+03	2.2188E+03	2.2193E+03	2.2253E+03	2.2530E+03	2.2192E+03	**2.2144E+03**
	Std	7.2939E+00	**3.3968E+00**	1.0124E+01	4.5642E+00	7.3177E+00	4.5630E+00	4.8236E+01	5.8485E+00	9.3215E+00
F9	Ave	2.5423E+03	**2.5244E+03**	2.5293E+03	2.5293E+03	2.5293E+03	2.5294E+03	2.6799E+03	2.5293E+03	2.5293E+03
	Std	3.7191E+01	1.5380E+01	5.1547E−03	6.2793E−06	6.6065E−04	1.8233E−01	5.6517E+01	3.1968E−03	**0.0000E+00**
F10	Ave	2.5279E+03	2.5467E+03	**2.5008E+03**	2.5112E+03	2.5086E+03	2.5617E+03	2.6135E+03	2.5575E+03	2.5373E+03
	Std	5.0233E+01	5.9832E+01	**2.7090E−01**	3.2390E+01	3.0953E+01	1.4894E+02	1.1558E+02	6.2210E+01	6.0672E+01
F11	Ave	2.8113E+03	2.7718E+03	2.7170E+03	**2.6100E+03**	2.6234E+03	2.7242E+03	3.0036E+03	2.7471E+03	2.7524E+03
	Std	1.8567E+02	1.4977E+02	1.1852E+02	**5.4770E+01**	8.0697E+01	1.6841E+02	1.8694E+02	1.8907E+02	1.6824E+02
F12	Ave	2.8640E+03	2.8756E+03	2.8665E+03	2.8638E+03	2.8641E+03	2.8643E+03	2.8716E+03	2.8655E+03	**2.8635E+03**
	Std	1.3011E+00	1.9165E+01	3.9517E+00	**1.2216E+00**	2.1883E+00	1.5989E+00	2.1309E+01	1.9789E+00	1.6657E+00

Significant values are in bold.

**Table 7:** Experimental outcomes for the CEC2022 benchmark (20-dimensional setting).

Function	Metric	VPPSO	IAGWO	IWOA	MadDE	SBO	AOO	HSO	RIME	MSRIME
F1	Ave	6.6758E+03	1.1458E+04	1.9260E+04	1.8514E+04	2.1395E+03	1.1030E+03	6.8671E+03	1.4764E+03	**4.3302E+02**
	Std	2.5934E+03	4.5513E+03	8.4137E+03	3.8634E+03	1.0914E+03	1.4005E+03	3.3185E+03	7.1283E+02	**1.4690E+02**
F2	Ave	4.7384E+02	4.9428E+02	4.6425E+02	4.5572E+02	4.5531E+02	4.5366E+02	5.6497E+02	4.5659E+02	**4.4901E+02**
	Std	3.0269E+01	2.5967E+01	2.1017E+01	**1.0759E+01**	1.9260E+01	2.0034E+01	5.1152E+01	1.1952E+01	1.3761E+01
F3	Ave	6.2742E+02	6.1200E+02	6.3972E+02	6.0188E+02	6.0272E+02	6.1883E+02	6.3880E+02	6.0574E+02	**6.0079E+02**
	Std	9.6431E+00	8.8884E+00	1.2853E+01	**5.7263E−01**	1.8706E+00	1.1078E+01	6.3121E+00	3.7251E+00	1.3721E+00
F4	Ave	8.6083E+02	8.7644E+02	8.9335E+02	8.6724E+02	8.6269E+02	8.5622E+02	9.1686E+02	8.6082E+02	**8.5214E+02**
	Std	1.4481E+01	1.6095E+01	1.3233E+01	**1.0931E+01**	1.5960E+01	2.0529E+01	1.1770E+01	2.6397E+01	3.1502E+01
F5	Ave	1.5445E+03	2.0472E+03	2.3329E+03	1.4944E+03	1.1441E+03	1.3274E+03	1.2639E+03	1.2286E+03	**9.2084E+02**
	Std	3.2892E+02	4.9758E+02	3.9712E+02	3.0553E+02	2.3671E+02	4.5591E+02	3.2732E+02	6.0021E+02	**7.0671E+01**
F6	Ave	4.8829E+03	**4.4220E+03**	7.8987E+03	4.1064E+04	5.5351E+03	7.9241E+03	4.5773E+03	1.1564E+04	9.6264E+03
	Std	**2.7887E+03**	2.8934E+03	6.9456E+03	3.6646E+04	5.3544E+03	6.5628E+03	3.4246E+03	7.4837E+03	8.1573E+03
F7	Ave	2.0955E+03	2.0773E+03	2.1188E+03	2.0656E+03	**2.0452E+03**	2.1121E+03	2.1374E+03	2.0976E+03	2.0505E+03
	Std	3.9818E+01	4.5714E+01	3.8695E+01	**1.2341E+01**	1.4226E+01	4.8843E+01	4.1375E+01	5.3463E+01	2.5245E+01
F8	Ave	2.2618E+03	2.2689E+03	2.2505E+03	2.2480E+03	2.2316E+03	2.2530E+03	2.4462E+03	2.2467E+03	**2.2269E+03**
	Std	6.3559E+01	6.5345E+01	4.1094E+01	**1.2467E+00**	2.0903E+01	4.4337E+01	9.2991E+01	4.0331E+01	4.6594E+01
F9	Ave	2.5128E+03	2.5086E+03	2.4811E+03	2.4810E+03	2.4833E+03	2.4832E+03	2.7161E+03	2.4816E+03	**2.4808E+03**
	Std	2.1895E+01	5.2084E+01	4.3901E−01	1.0503E−01	2.3427E+00	3.0851E+00	1.0900E+02	4.4855E−01	**2.9212E−05**
F10	Ave	3.2289E+03	2.7714E+03	2.7713E+03	**2.5305E+03**	2.5333E+03	3.4678E+03	3.7577E+03	2.8518E+03	2.9026E+03
	Std	9.5173E+02	1.8801E+02	6.1791E+02	7.1334E+01	**6.6565E+01**	7.8404E+02	7.0238E+02	2.7089E+02	3.4866E+02
F11	Ave	3.0120E+03	2.9381E+03	2.9251E+03	**2.9134E+03**	2.9441E+03	2.9434E+03	3.6329E+03	2.9330E+03	2.9284E+03
	Std	3.0563E+02	1.1807E+02	1.0389E+02	**4.3550E+01**	1.1347E+02	9.8971E+01	3.6219E+02	1.1180E+02	1.0847E+02
F12	Ave	2.9855E+03	3.0023E+03	3.0493E+03	2.9603E+03	2.9660E+03	2.9822E+03	2.9958E+03	2.9685E+03	**2.9498E+03**
	Std	3.2663E+01	1.5605E+02	3.5260E+01	**6.9966E+00**	1.7281E+01	5.1781E+01	2.7059E+01	2.3540E+01	9.3797E+00

Significant values are in bold.

In low-dimensional scenarios (CEC2017-30D, CEC2022-10D), MSRIME demonstrates significant advantages on most functions. As shown in Table 4, for the F1 function of CEC2017-30D, MSRIME achieves an average fitness of 5.1720E+03, which is only 0.13% of the original RIME (3.8513E+06) and outperforms HSO (6.9748E+03) and AOO (7.3666E+04). For F14, MSRIME’s average fitness is 1.5684E+03, far lower than the original RIME (9.3913E+04) and MadDE (1.1320E+05), indicating that it can accurately locate the optimal solution for both low-dimensional unimodal and multimodal problems.

As the dimensionality increases to 100D (CEC2017-100D), the performance of most algorithms deteriorates significantly, while MSRIME maintains stable superiority. For instance, in F5, MSRIME achieves an average fitness of 1.0495E+03, which is 19.4% lower than RIME (1.3022E+03) and 28.9% lower than IWOA (1.4767E+03). On the high-dimensional complex function F13, MSRIME achieves 1.0707E+04, only 0.48% of RIME’s value (2.2309E+06), and the standard deviation (5.0643E+03) is significantly lower than that of other algorithms, verifying its ability to resist the “curse of dimensionality” in high-dimensional search spaces.

On the CEC2022 benchmark set, MSRIME’s advantages are similarly evident. As shown in Table 6 (CEC2022-10D), for F1, MSRIME achieves an average fitness of 3.0000E+02 with a standard deviation of only 1.9269E−06, reaching the theoretical optimum, whereas original RIME (3.0068E+02) and IAGWO (3.5437E+02) exhibit noticeable errors. In Table 7 (CEC2022-20D), for F5, MSRIME achieves 9.2084E+02, which is 25.1% lower than RIME (1.2286E+03) and 55.0% lower than IAGWO (2.0472E+03), further demonstrating its strong adaptability across different test sets and dimensionalities.

The standard deviation (Std) reflects the optimization stability of the algorithms. MSRIME consistently exhibits lower Std values across benchmark functions. For example, in F4 of CEC2017-30D, MSRIME’s Std is 2.2456E+01, lower than RIME (3.6875E+01) and VPPSO (3.0792E+01); in F25 of CEC2017-100D, its Std is 1.0691E+01, only 33.8% of RIME’s value (3.1613E+01), indicating that MSRIME maintains stable optimization performance over multiple independent runs and is less affected by initial population and iterative fluctuations. In F9 of CEC2022-10D, MSRIME achieves zero fluctuation with a Std of 0.0000E+00, while HSO (5.6517E+01) and IAGWO (1.5380E+01) exhibit significantly higher Std values, further demonstrating MSRIME’s robustness in complex optimization scenarios.

Figure 4 illustrates the dynamic convergence process of the compared algorithms. In the CEC2017-30D functions F1, F9, and F14, the convergence curves of MSRIME consistently lie at the bottom, and within the first 50 iterations, the fitness values drop rapidly, quickly approaching the optimal solution region. Taking F14 as an example, MSRIME reaches stable convergence at iteration 100, whereas the original RIME and HSO require over 300 iterations to stabilize, with final fitness values significantly higher than MSRIME.

In high-dimensional scenarios (CEC2017-100D, functions F1, F8, F30), MSRIME’s convergence advantage is even more pronounced. For F30, the convergence curve shows that MSRIME achieves a fitness value of 1.0039E+05 at iteration 200, while the original RIME (8.2300E+05) and IWOA (1.1766E+05) remain at high levels with slower decrease rates. This demonstrates that MSRIME’s adaptive differential mutation factor combined with the multi-strategy cooperative mechanism can effectively balance global exploration and local exploitation in high-dimensional search spaces, avoiding convergence stagnation.

On the CEC2022 test set (functions F1, F6, F12), MSRIME also exhibits the characteristics of rapid decline and early stabilization. For F6, MSRIME’s fitness falls below 1.9338E+03 at iteration 150, while the original RIME (3.7986E+03) and VPPSO (4.5589E+03) remain above 2.0000E+03, further confirming its efficient convergence across different benchmark functions.

In summary, whether in low-dimensional or high-dimensional scenarios, and across both CEC2017 and CEC2022 test sets, MSRIME consistently outperforms the original RIME and other mainstream algorithms in optimization accuracy, stability, and convergence speed. The multi-strategy integration enables effective adaptation to problems of varying complexity, providing a robust algorithmic foundation for practical applications such as multi-level threshold image segmentation and robot path planning.

### Statistical evaluation via Friedman test

Establishing a comprehensive performance ranking for the MSRIME technique necessitates statistical tools capable of assessing multiple interrelated methods concurrently. Tailored to this purpose, the Friedman procedure serves as a non-parametric approach for comparing various algorithms based on their ordinal positions across a series of datasets. This framework inherently circumvents any assumptions regarding the underlying distribution of the measured outcomes. Such a characteristic renders it particularly potent for comparative analyses involving numerous algorithms evaluated against an identical collection of benchmark functions. The test statistic is computed using the following expression^61,62^:

12 $$\begin{array}{*{20}c} {Q = \frac{12}{{kn\left( {k + 1} \right)}}\mathop \sum \limits_{j = 1}^{k} R_{j}^{2} - 3n\left( {k + 1} \right)} \\ \end{array}$$

Within this formulation, $n$ denotes the quantity of blocks, $k$ signifies the number of treatments, and $R_{j}$ stands for the cumulative rank of the *j*th. treatment. Assuming the sample sizes for $n$ and $k$ meet minimum thresholds, the derived $Q$ statistic adheres to a $\chi^{2}$ probability distribution possessing $k - 1$ degrees of freedom^62^.

Table 8 clearly presents the rankings of nine algorithms across four scenarios: CEC2017 (30D, 100D) and CEC2022 (10D, 20D). MSRIME consistently achieves first place in all scenarios, with its mean ranking (M.R) significantly lower than that of other algorithms. In the CEC2017-30D scenario, MSRIME’s mean ranking is 1.93, only 53.6% of the second-ranked SBO (3.60), and substantially lower than the original RIME (5.07) and IWOA (6.93). In the high-dimensional scenario (CEC2017-100D), MSRIME’s mean ranking further decreases to 1.77, whereas most comparative algorithms experience rank increases, e.g., MadDE rises from 4.67 to 7.43 and IWOA from 6.93 to 6.43. This indicates that high-dimensional environments significantly impair the performance of other algorithms, while MSRIME maintains a stable advantage due to its multi-strategy adaptive mechanism.

**Table 8:** Friedman test ranking summary.

Suites	CEC2017				CEC2022			
Dimensions	30		100		10		20	
Algorithms	*M.R*	*T.R*	*M.R*	*T.R*	*M.R*	*T.R*	*M.R*	*T.R*
VPPSO	6.43	8	5.37	5	5.75	6	5.92	7
IAGWO	5.70	6	4.43	3	5.83	7	5.50	6
IWOA	6.93	9	6.43	8	6.42	8	6.50	8
MadDE	4.67	4	7.43	9	2.83	2	3.92	3
SBO	3.60	2	5.47	6	3.17	3	3.58	2
AOO	4.43	3	3.43	2	5.33	4	4.75	4
HSO	6.23	7	5.53	7	8.58	9	7.83	9
RIME	5.07	5	5.13	4	5.42	5	4.83	5
MSRIME	1.93	1	1.77	1	1.67	1	2.17	1

On the CEC2022 benchmark, MSRIME’s superiority remains evident. In the CEC2022-10D scenario, its mean ranking is 1.67, with a clear gap from the second-ranked MadDE (2.83) and third-ranked SBO (3.17). In the CEC2022-20D scenario, MSRIME’s mean ranking is 2.17, still leading SBO (3.58) and outperforming the original RIME (4.83) by 2.66 ranking units. From the total ranking (T.R) perspective, MSRIME consistently occupies the first position in all four scenarios, whereas other algorithms exhibit considerable rank fluctuations. For example, AOO ranks 2nd in CEC2017-100D (M.R = 3.43) but drops to 4th in CEC2022-10D (M.R = 5.33), further highlighting MSRIME’s stability and adaptability across diverse scenarios.

Examining the ranking gaps, the lowest-performing algorithms (e.g., HSO, IWOA) show maximum mean ranking differences of up to 7.21 (e.g., HSO in CEC2022-10D with M.R = 8.58 versus MSRIME), and these algorithms generally perform worse in high-dimensional or complex benchmark sets such as CEC2022, reflecting their limitations in handling diverse optimization problems. In contrast, MSRIME’s mean ranking fluctuates minimally across the four scenarios (1.67 ~ 2.17), demonstrating the strong robustness of the algorithm across varying dimensions and benchmark sets.

**Fig. 5:**
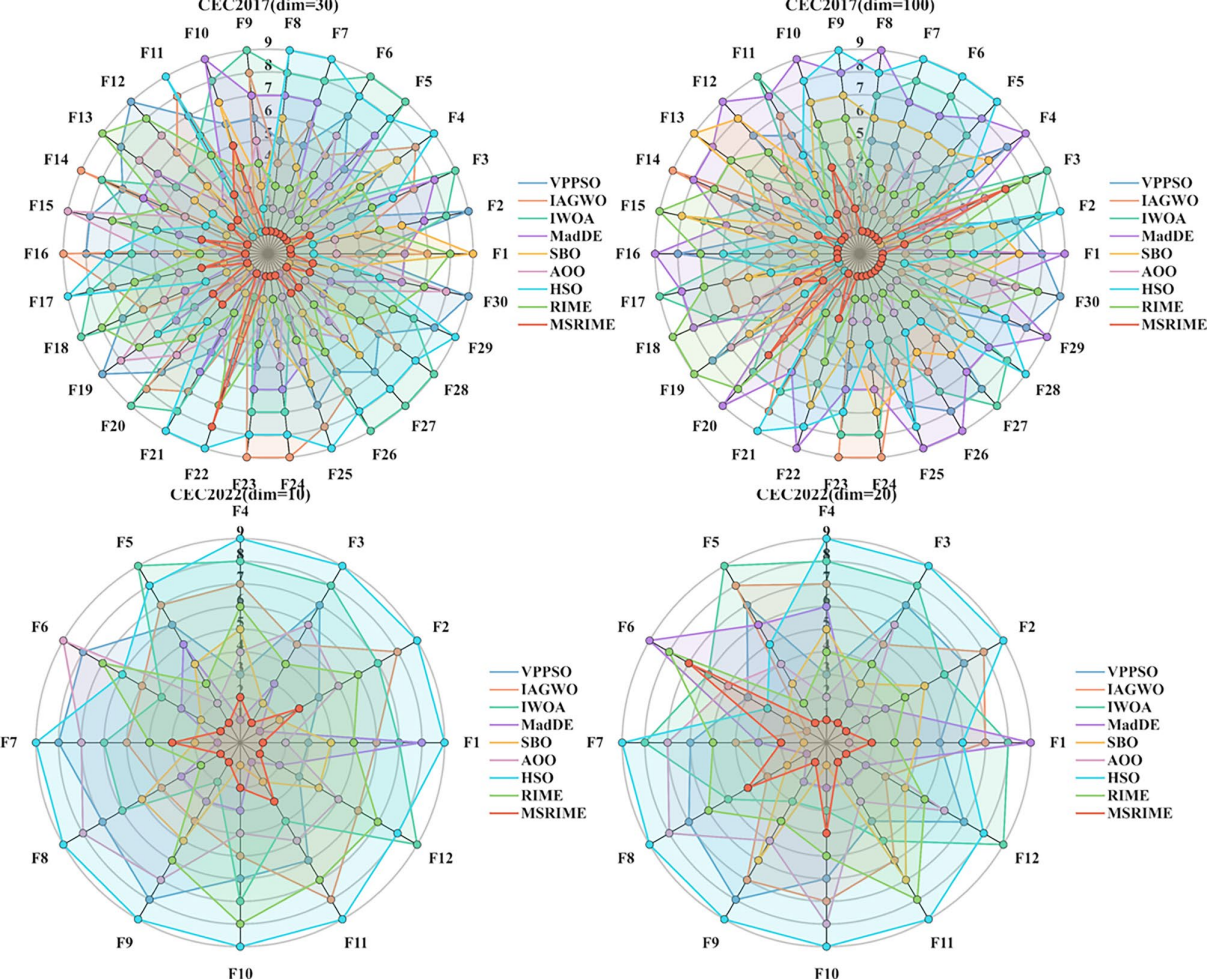
Visualization of the relative ranking spread over the collected algorithmic set.

Figure 5 presents the ranking distribution of each algorithm across different functions in the form of a heatmap, where darker colors indicate better rankings (smaller values). The heatmap clearly shows that MSRIME exhibits a “globally dark” pattern, maintaining top or near-top rankings across nearly all functions (F1 ~ F30), whereas other algorithms only perform well on certain functions, showing clear “local advantage” limitations. For example, SBO achieves favorable rankings (darker colors) on F1 ~ F5 in CEC2017-30D but drops significantly on complex functions such as F12 ~ F15 (lighter colors). Similarly, MadDE performs well on F6 ~ F10 in CEC2022-10D but ranks at the bottom for F25 ~ F30 in CEC2017-100D.

From the perspective of function types, MSRIME maintains excellent rankings on unimodal functions (F1 ~ F5), multimodal functions (F6 ~ F15), hybrid functions (F16 ~ F25), and composite functions (F26 ~ F30). Its ranking advantage is particularly pronounced on high-dimensional multimodal functions (e.g., F13 ~ F15 in CEC2017-100D), whereas the original RIME and other comparative algorithms generally show ranking degradation on these functions. This highlights that MSRIME’s multi-strategy cooperative mechanism can effectively address the optimization requirements of diverse function types and prevent performance fluctuations caused by variations in function characteristics.

Furthermore, MSRIME’s heatmap shows no evident “light-colored regions,” indicating that there are no functions where its performance is weak. In contrast, other algorithms exhibit clear weaknesses: HSO consistently ranks lower across F1 ~ F30 in all scenarios (lighter colors), and IAGWO experiences significant ranking drops in high-dimensional functions. This further confirms MSRIME’s comprehensive performance; the integration of adaptive differential mutation, multi-strategy pool, and probability-based selection enables robust coverage across diverse optimization scenarios.

In summary, both the Friedman test results and the ranking heatmap demonstrate that MSRIME exhibits significant and stable overall performance advantages across different benchmark sets, dimensionalities, and function types. The ranking results are statistically significant, providing quantitative evidence of the algorithm’s superiority and offering important guidance for algorithm selection in practical applications.

### Computational time analysis

**Fig. 6:**
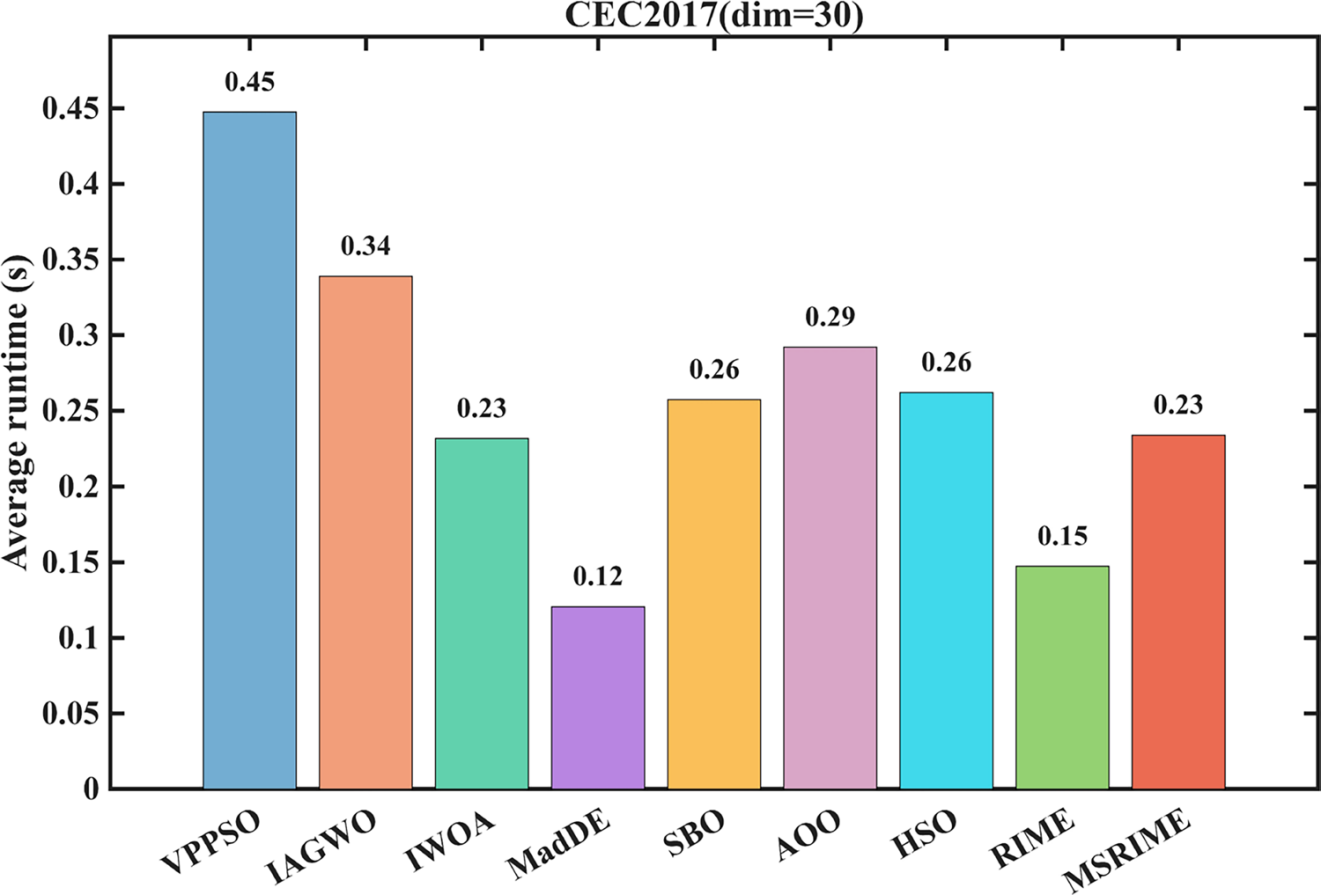
Visualization of the relative ranking spread over the collected algorithmic set.

To further evaluate the computational efficiency of the proposed algorithm, the average runtime of MSRIME and the compared algorithms on the CEC2017 (30-dimensional) benchmark is presented in Fig. 6.

As shown in Fig. 5, the runtime of different algorithms varies significantly. VPPSO exhibits the highest computational cost (0.45 s), followed by IAGWO (0.34 s) and AOO (0.29 s), indicating relatively higher computational overhead due to their complex update mechanisms. In contrast, MadDE achieves the lowest runtime (0.12 s), followed by the original RIME (0.15 s), demonstrating the advantage of simpler algorithmic structures.

The proposed MSRIME achieves an average runtime of approximately 0.23 s, which is comparable to IWOA (0.23 s) and lower than several algorithms such as SBO (0.26 s), HSO (0.26 s), and AOO (0.29 s). Although MSRIME introduces multiple enhancement strategies, including adaptive mutation factor adjustment, multi-strategy collaborative updates, and probability-based strategy selection, its computational cost remains within a moderate range.

Compared with the original RIME, MSRIME incurs a certain increase in runtime due to additional strategy evaluation and selection operations. However, this increase is limited and remains acceptable considering the significant improvements in optimization accuracy, convergence speed, and robustness demonstrated in previous sections.

Overall, MSRIME achieves a favorable trade-off between computational cost and optimization performance. The results indicate that the proposed multi-strategy enhancement framework effectively improves solution quality without introducing excessive computational overhead, making it suitable for practical optimization and image processing applications.

## Multilevel thresholding image segmentation

To verify the practical performance of the Multi-strategy Self-adaptive Rime Optimization Algorithm (MSRIME) in multi-level threshold image segmentation tasks, this chapter designs comparative experiments. Using Otsu’s maximum between-class variance method as the objective function, segmentation tests are conducted on multiple benchmark images at different threshold levels. The segmentation results are quantified and analyzed using multi-dimensional evaluation metrics.

### Evaluation metrics

Three types of metrics—Peak Signal-to-Noise Ratio (PSNR), Structural Similarity (SSIM), and Feature Similarity (FSIM)—are used to assess the segmentation quality, defined as follows^5^:

(1) Peak Signal-to-Noise Ratio (PSNR)

PSNR measures the distortion between the segmented image and the original image. A higher PSNR indicates lower distortion and better image quality. It is computed as:

13 $$\begin{aligned} & PSNR = 10 \times {\mathrm{log}}_{10} \left( {\frac{{255^{2} }}{{{\mathrm{MSE}}}}} \right) \\ & MSE = \frac{1}{M \times N}\sum\limits_{j = 1}^{M} {\sum\limits_{k = 1}^{N} {} \left[ {I\left( {j,k} \right) - I^{\prime } \left( {j,k} \right)} \right]^{2} } \\ \end{aligned}$$

where $M \times N$ represents the image dimensions, $I\left( {j,k} \right)$ is the grayscale value of the original image at coordinate $\left( {j,k} \right)$, and $I^{\prime}\left( {j,k} \right)$ is the grayscale value of the segmented image at the same coordinate.

(2) Structural Similarity (SSIM)

SSIM evaluates the similarity between two images from luminance, contrast, and structure dimensions. Its value ranges from [− 1,1], with values closer to 1 indicating better structural preservation of the segmented image relative to the original image. The computation is as follows^63^:

14 $$\begin{aligned} & {\mathrm{SSIM}}\left( {I,I^{\prime } } \right) = \frac{{\left( {2\mu_{I} \mu_{{I^{\prime } }} + C_{1} } \right)\left( {2\sigma_{{II^{\prime } }} + C_{2} } \right)}}{{\left( {\mu_{I}^{2} + \mu_{{I^{\prime } }}^{2} + C_{1} } \right)\left( {\sigma_{I}^{2} + \sigma_{{I^{\prime } }}^{2} + C_{2} } \right)}} \\ & C_{1} = \left( {K_{1} \times L} \right)^{2} ,C_{2} = \left( {K_{2} \times L} \right)^{2} \\ \end{aligned}$$

where $\mu_{I} ,\mu_{{I^{\prime}}}$ are the mean grayscale values of the original image $I$ and segmented image $I^{\prime }$, respectively, $\sigma_{I}^{2} ,\sigma_{{I^{\prime}}}^{2}$ are the variances of $I$ and $I^{\prime }$, $\sigma_{{II^{\prime } }}$ is the covariance between $I$ and $I^{\prime}$. To avoid division by zero, constants $K_{1} = 0.01$ and $K_{2} = 0.03$ are used, and $L$ represents the maximum grayscale level of the image (typically 255).

(3) Feature Similarity (FSIM)

FSIM evaluates the feature-preserving capability of an image by combining phase congruency and gradient magnitude information. Higher values indicate smaller segmentation errors. Its computation is defined as^63^:

15 $$\begin{aligned} & {\mathrm{FSIM}}\left( {I,I^{\prime } } \right) = \frac{{\mathop \sum \nolimits_{x} \left[ {{\mathrm{PC}}_{m} \left( x \right) \times S_{L} \left( x \right)} \right]}}{{\mathop \sum \nolimits_{x} {\mathrm{PC}}_{m} \left( x \right)}} \\ & {\mathrm{PC}}_{m} \left( x \right) = \max \left( {{\mathrm{PC}}_{I} \left( x \right),{\mathrm{PC}}_{{I^{\prime}}} \left( x \right)} \right) \\ \end{aligned}$$

where ${\mathrm{PC}}_{I} \left( x \right)$ and ${\mathrm{PC}}_{{I^{\prime}}} \left( x \right)$ represent the phase congruency of the original image $I$ and the segmented image $I^{\prime}$, respectively.

16 $$\begin{aligned} & S_{L} \left( x \right) = \alpha \times S_{PC} \left( x \right) + \left( {1 - \alpha } \right) \times S_{GM} \left( x \right),\;\alpha = \beta = 1 \\ & S_{PC} \left( x \right) = \frac{{2{\mathrm{PC}}_{I} \left( x \right){\mathrm{PC}}_{{I^{\prime } }} \left( x \right) + T_{1} }}{{{\mathrm{PC}}_{I} (x)^{2} + {\mathrm{PC}}_{{I^{\prime } }} (x)^{2} + T_{1} }} \\ & S_{GM} \left( x \right) = \frac{{2{\mathrm{GM}}_{I} \left( x \right){\mathrm{GM}}_{{I^{\prime } }} \left( x \right) + T_{2} }}{{{\mathrm{GM}}_{I} (x)^{2} + {\mathrm{GM}}_{{I^{\prime } }} (x)^{2} + T_{2} }} \\ \end{aligned}$$

where $T_{1}$ and $T_{2}$ are constants used to enhance numerical stability.

### Experimental design

To fairly evaluate the performance of the proposed algorithm, eight mainstream population-based intelligence algorithms, including Particle Swarm Optimization (PSO), Grey Wolf Optimizer (GWO), and Whale Optimization Algorithm (WOA), were selected as comparison methods. For all algorithms, the population size was set to $N = 30$ and the maximum number of iterations to $T = 100$; other parameters were set according to their respective original references.

**Fig. 7:**
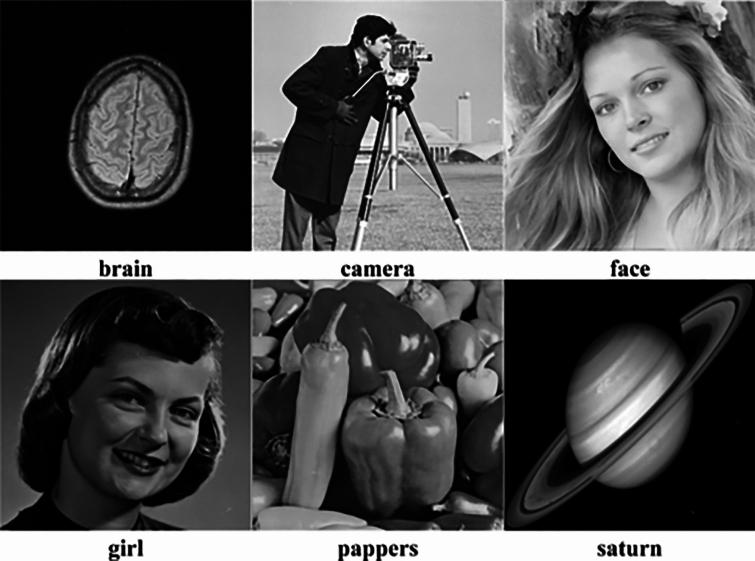
Baseline images of different styles.

Six benchmark images with diverse styles (brain, camera, face, girl, pappers, and saturn) were employed (as shown in Fig. 7) to cover a variety of texture complexities and gray-level distributions. These images are obtained from a publicly available open-source repository (https://gitcode.com/open-source-toolkit/5870e), which is commonly used for benchmarking in image processing research. For different segmentation precision requirements, four threshold levels—4, 6, 8, and 10—were tested.

To evaluate the optimization accuracy and stability of each algorithm, each experiment was independently repeated 30 times, and the mean (Mean) and standard deviation (Std) of the objective function, PSNR, SSIM, and FSIM were recorded.

All images used in Fig. 7 are standard benchmark images widely used in the image processing community and obtained from publicly available datasets. These images are distributed for research purposes, and no personal or sensitive information is involved. Therefore, permission for academic publication under the journal’s license is not required.

In this study, the Otsu maximum between-class variance method is adopted as the objective function for threshold optimization. Its core principle is to determine the optimal thresholds by maximizing the between-class variance.

For an image with $L$ gray levels, the probability of a pixel with gray level $i$ is:

17 $$\begin{array}{*{20}c} {P_{i} = \frac{{n_{i} }}{N}} \\ \end{array}$$

where $n_{i}$ is the number of pixels with gray level $i$, and $N = \sum\nolimits_{i = 0}^{L - 1} {n_{i} }$ is the total number of pixels. It satisfies $P_{i} \ge 0,{ }and{ }P_{0} + P_{1} + \cdots + P_{L - 1} = 1$.

18 $$\begin{array}{*{20}c} {\omega_{i} = \mathop \sum \limits_{{j = T_{i - 1} + 1}}^{{T_{i} }} P_{j} \left( {T_{0} = - 1,T_{k + 1} = L - 1} \right)} \\ \end{array}$$

19 $$\begin{array}{*{20}c} {\mu_{i} = \frac{1}{{\omega_{i} }}\mathop \sum \limits_{{j = T_{i - 1} + 1}}^{{T_{i} }} j \times P_{j} } \\ \end{array}$$

20 $$\begin{array}{*{20}c} {\mu = \mathop \sum \limits_{i = 0}^{k} \omega_{i} \mu_{i} } \\ \end{array}$$

21 $$\begin{array}{*{20}c} {\sigma^{2} = \mathop \sum \limits_{i = 0}^{k} \omega_{i} \left( {\mu_{i} - \mu } \right)^{2} } \\ \end{array}$$

The optimal threshold combination $T_{{\text{best }}} = \left( {T_{1}^{*} ,T_{2}^{*} , \ldots ,T_{k}^{*} } \right)$ is the set of thresholds that maximizes the between-class variance:

22 $$\begin{array}{*{20}c} {T_{{\text{best }}} = arg\mathop {max}\limits_{{T_{1} ,T_{2} , \ldots ,T_{k} }} \sigma^{2} } \\ \end{array}$$

### Experimental results and analysis

The MSRIME algorithm was applied to perform multi-level thresholding on five selected images, using the Otsu criterion as the optimization objective. In Otsu-based thresholding, the objective function value, as well as the evaluation metrics PSNR, FSIM, and SSIM, can effectively measure the performance of a segmentation algorithm. Higher values of these metrics indicate better segmentation quality.

The experimental results demonstrate that the proposed MSRIME algorithm outperforms all comparative algorithms in terms of the objective function value, PSNR, FSIM, and SSIM. Table 9 shows the distribution of the optimal thresholds obtained by MSRIME on the image histograms. Table 10 reports the mean and standard deviation of the optimal fitness values achieved by each algorithm under the Otsu objective, along with their average rankings. Tables 11, 12 and 13 summarize the mean, standard deviation, and average ranking of PSNR, FSIM, and SSIM, respectively, for each algorithm under the Otsu objective function.

**Table 9:**
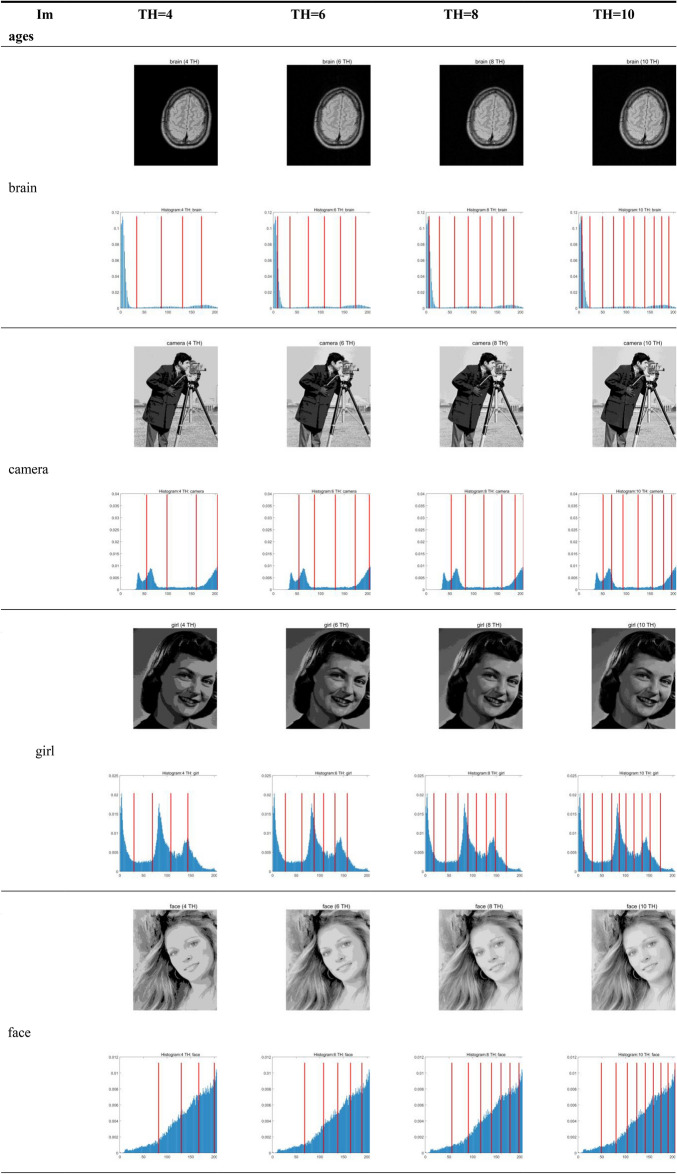

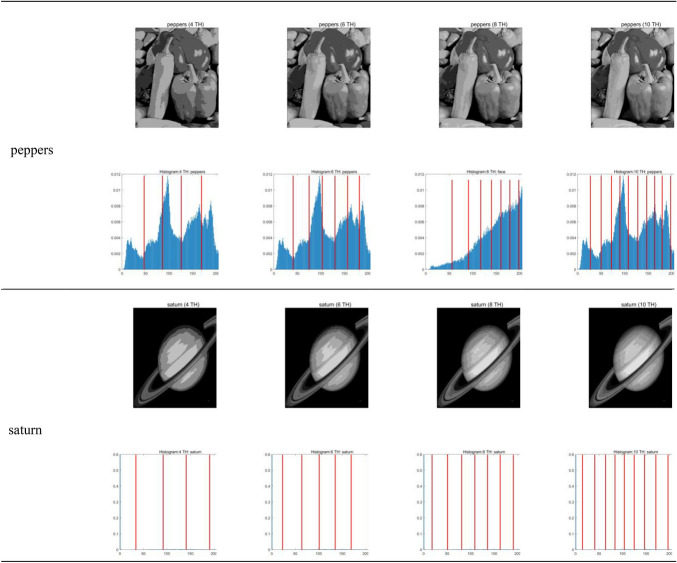
Multi-level threshold segmentation outcomes employing Otsu’s method as the objective function.

**Table 10:** Mean and Standard Deviation for optimal Otsu-based fitness function values.

Images	TH	Metrics	VPPSO	IAGWO	IWOA	MadDE	SBO	AOO	HSO	RIME	MSRIME
Brian	4	Ave	3.7305E+03	3.7305E+03	3.7305E+03	3.7303E+03	3.7305E+03	3.7305E+03	3.7142E+03	3.7305E+03	**3.7305E+03**
		Std	1.3585E−01	2.3181E−01	3.2160E−02	1.7549E−01	6.5825E−02	5.7614E−02	8.4215E+00	4.8910E−02	**1.2606E−02**
	6	Ave	3.7507E+03	3.7503E+03	3.7508E+03	3.7507E+03	3.7508E+03	3.7509E+03	3.7379E+03	3.7508E+03	**3.7509E+03**
		Std	4.9555E−01	8.5902E−01	5.8631E−01	5.6122E−01	5.9683E−01	6.3341E−01	4.7407E+00	5.9479E−01	**3.9803E−01**
	8	Ave	3.7623E+03	3.7630E+03	3.7628E+03	3.7624E+03	3.7630E+03	3.7630E+03	3.7494E+03	3.7631E+03	**3.7636E+03**
		Std	1.8150E+00	1.9850E+00	1.5015E+00	5.7648E−01	1.0674E+00	8.2356E−01	4.8882E+00	1.4532E+00	**2.4976E−01**
	10	Ave	3.7688E+03	3.7688E+03	3.7688E+03	3.7681E+03	3.7685E+03	3.7685E+03	3.7556E+03	3.7690E+03	**3.7694E+03**
		Std	6.6347E−01	9.3689E−01	9.5955E−01	4.8285E−01	9.7967E−01	8.9405E−01	3.1499E+00	3.3012E−01	**1.0586E−01**
Camera	4	Ave	4.5998E+03	4.5996E+03	4.5998E+03	4.6005E+03	4.5999E+03	4.5998E+03	4.5816E+03	4.6002E+03	**4.6009E+03**
		Std	1.1784E+00	1.3399E+00	1.0965E+00	**4.6487E−01**	1.0551E+00	1.2125E+00	1.0965E+01	1.1105E+00	6.1244E−01
	6	Ave	4.6511E+03	4.6506E+03	4.6517E+03	4.6495E+03	4.6512E+03	4.6511E+03	4.6188E+03	4.6515E+03	**4.6517E+03**
		Std	1.9689E+00	2.8211E+00	1.9763E−02	1.3995E+00	1.4206E+00	7.5363E−01	8.7746E+00	2.2175E−01	**4.3726E−03**
	8	Ave	4.6693E+03	4.6688E+03	4.6698E+03	4.6670E+03	4.6691E+03	4.6684E+03	4.6390E+03	4.6697E+03	**4.6705E+03**
		Std	1.0037E+00	1.7070E+00	7.8656E−01	8.6758E−01	1.0777E+00	1.1254E+00	8.6887E+00	8.0367E−01	**4.2254E−01**
	10	Ave	4.6798E+03	4.6788E+03	4.6804E+03	4.6774E+03	4.6790E+03	4.6788E+03	4.6533E+03	4.6804E+03	**4.6808E+03**
		Std	1.0802E+00	2.4047E+00	8.1145E−01	1.1933E+00	1.8322E+00	2.0885E+00	7.3178E+00	4.5482E−01	**1.0646E−01**
Girl	4	Ave	2.1225E+03	2.1221E+03	2.1225E+03	2.1219E+03	2.1224E+03	2.1223E+03	2.0919E+03	2.1224E+03	**2.1225E+03**
		Std	1.6971E−02	7.2909E−01	1.8124E−02	4.0016E−01	2.1186E−01	1.3954E−01	1.5613E+01	4.0797E−02	**6.2733E−03**
	6	Ave	2.1849E+03	2.1830E+03	2.1849E+03	2.1828E+03	2.1846E+03	2.1841E+03	2.1486E+03	2.1849E+03	**2.1850E+03**
		Std	2.3254E−01	4.2530E+00	4.0771E−01	1.2765E+00	7.9929E−01	1.1507E+00	1.2612E+01	1.8691E−01	**1.1877E−02**
	8	Ave	2.2107E+03	2.2082E+03	2.2107E+03	2.2078E+03	2.2101E+03	2.2076E+03	2.1807E+03	2.2109E+03	**2.2112E+03**
		Std	5.7714E−01	3.7571E+00	6.4634E−01	1.3028E+00	1.2937E+00	4.0021E+00	9.9701E+00	3.0479E−01	**1.1121E−01**
	10	Ave	2.2234E+03	2.2218E+03	2.2234E+03	2.2210E+03	2.2230E+03	2.2208E+03	2.1979E+03	2.2240E+03	**2.2246E+03**
		Std	1.0243E+00	2.2528E+00	1.9617E+00	1.2652E+00	1.2893E+00	3.5830E+00	7.6573E+00	7.4927E−01	**1.6052E−01**
Face	4	Ave	2.5339E+03	2.5340E+03	2.5340E+03	2.5336E+03	2.5339E+03	2.5339E+03	2.5058E+03	2.5339E+03	**2.5340E+03**
		Std	1.1990E−02	6.3516E−03	6.2652E−E−03	2.8626E−01	1.9449E−01	4.0094E−02	1.4030E+01	5.7705E−02	**5.1526E−03**
	6	Ave	2.5846E+03	2.5842E+03	2.5846E+03	2.5832E+03	2.5844E+03	2.5842E+03	2.5500E+03	2.5845E+03	**2.5847E+03**
		Std	2.5357E−01	1.1394E+00	2.4079E−01	6.0205E−01	4.1550E−01	4.8432E−01	1.5162E+01	2.7242E−01	**1.8945E−01**
	8	Ave	2.6068E+03	2.6060E+03	2.6064E+03	2.6035E+03	2.6058E+03	2.6053E+03	2.5778E+03	2.6068E+03	**2.6072E+03**
		Std	8.5129E−01	2.3028E+00	1.1395E+00	1.1207E+00	1.8739E+00	1.5815E+00	1.1305E+01	3.8083E−01	**3.8934E−02**
	10	Ave	2.6172E+03	2.6163E+03	2.6168E+03	2.6147E+03	2.6162E+03	2.6169E+03	2.4177E+03	2.6174E+03	**2.6178E+03**
		Std	7.6516E−01	2.1579E+00	1.5544E+00	9.9978E−01	1.6015E+00	8.5570E−01	6.5726E+02	3.8571E−01	**1.6420E−01**
Peppers	4	Ave	2.7011E+03	2.7007E+03	2.7006E+03	2.7004E+03	2.7010E+03	2.7006E+03	2.6758E+03	2.7011E+03	**2.7011E+03**
		Std	2.4518E−02	1.3571E+00	1.5554E+00	5.8724E−01	6.6702E−01	1.3766E+00	1.4308E+01	6.2050E−02	**3.8969E−04**
	6	Ave	2.7688E+03	2.7685E+03	2.7689E+03	2.7663E+03	2.7686E+03	2.7686E+03	2.7373E+03	2.7687E+03	**2.7690E+03**
		Std	4.9498E−01	1.2165E+00	2.5403E−01	1.3152E+00	7.7593E−01	7.2084E−01	1.1003E+01	3.2736E−01	**9.0789E−04**
	8	Ave	2.7954E+03	2.7940E+03	2.7944E+03	2.7911E+03	2.7943E+03	2.7929E+03	2.7632E+03	2.7953E+03	**2.7957E+03**
		Std	3.6562E−01	2.5594E+00	1.9587E+00	1.9166E+00	1.7293E+00	2.8634E+00	7.2786E+00	3.9125E−01	**3.0001E−02**
	10	Ave	2.8075E+03	2.8066E+03	2.8080E+03	2.8044E+03	2.8067E+03	2.8062E+03	2.7804E+03	2.8080E+03	**2.8086E+03**
		Std	9.5718E−01	2.4370E+00	9.5181E−01	1.1590E+00	1.9545E+00	1.7019E+00	9.1448E+00	7.4459E−01	**4.0268E−01**
Saturn	4	Ave	5.2220E+03	5.2210E+03	5.2220E+03	5.2217E+03	5.2219E+03	5.2219E+03	5.1940E+03	5.2220E+03	**5.2220E+03**
		Std	5.7652E−03	4.5421E+00	2.4468E−03	3.2307E−01	1.6607E−01	3.7104E−02	1.7287E+01	2.6045E−02	**9.2504E−13**
	6	Ave	5.2730E+03	5.2716E+03	5.2724E+03	5.2719E+03	5.2726E+03	5.2728E+03	5.2473E+03	5.2730E+03	**5.2731E+03**
		Std	2.9767E−02	2.2682E+00	2.1510E+00	5.4492E−01	1.3719E+00	2.4352E−01	1.0429E+01	6.0578E−02	**2.7118E−03**
	8	Ave	5.2936E+03	5.2926E+03	5.2932E+03	5.2920E+03	5.2932E+03	5.2930E+03	5.2746E+03	5.2936E+03	**5.2939E+03**
		Std	2.5895E−01	1.5408E+00	1.2842E+00	6.7790E−01	7.6815E−01	6.0821E−01	8.1209E+00	3.1199E−01	**1.3519E−02**
	10	Ave	5.3036E+03	5.3025E+03	5.3030E+03	5.3017E+03	5.3028E+03	5.3031E+03	5.2854E+03	5.3034E+03	**5.3037E+03**
Friedman-rank			2.84	5.27	3.36	7.46	4.90	5.52	8.99	4.38	**2.28**
Final-rank			2	6	3	8	5	7	9	4	**1**

Significant values are in bold.

**Table 11:** Results of the PSNR values using Otsu’s method.

Images	TH	Metrics	VPPSO	IAGWO	IWOA	MadDE	SBO	AOO	HSO	RIME	MSRIME
Brian	4	Ave	25.0656	25.0599	25.0861	25.0661	25.0689	25.0991	24.2980	**25.1047**	25.0594
		Std	**0.0530**	0.0588	0.0626	0.1037	0.0600	0.0627	0.6541	0.0736	0.0953
	6	Ave	27.4925	27.4244	27.5011	27.1535	27.4864	27.4260	26.0718	27.4941	**27.5202**
		Std	0.1884	0.2797	0.1900	0.2006	0.1968	0.1943	0.6724	0.1968	**0.1815**
	8	Ave	29.3211	29.3493	29.3283	29.1231	29.3636	29.3029	27.3187	29.3503	**29.4704**
		Std	0.1799	0.3700	0.3463	0.2777	0.2366	0.2644	0.8595	0.3320	**0.1467**
	10	Ave	30.8317	30.7707	30.8219	30.5527	30.7330	30.6562	28.2545	30.8088	**31.0253**
		Std	0.2645	0.3581	0.3456	0.3242	0.3955	0.4450	0.6698	0.2411	**0.1173**
Camera	4	Ave	19.0010	18.8424	18.7602	19.6897	19.0125	18.8501	17.5578	19.1182	**19.7868**
		Std	1.0034	0.9204	0.9299	0.5542	0.9097	0.9659	0.4323	0.9328	**0.1957**
	6	Ave	21.8845	21.8295	**21.9239**	21.8018	21.8829	21.8892	19.5192	21.8988	21.9158
		Std	0.1513	0.2681	**0.0306**	0.2913	0.1348	0.1351	1.3032	0.0984	0.0379
	8	Ave	23.1972	23.1881	23.1817	22.9340	23.1796	23.0238	20.9334	23.1220	**23.3945**
		Std	0.2505	0.3056	0.2220	0.4928	0.3245	0.3867	1.4195	0.2623	**0.1253**
	10	Ave	24.0489	24.3510	**24.4581**	24.0047	24.1318	24.4514	22.1884	24.2879	24.3795
		Std	0.4436	0.4995	0.3632	0.6029	0.5802	0.6395	1.7330	0.3624	**0.3353**
Girl	4	Ave	19.7519	19.7220	19.7519	19.6937	19.7461	19.7318	18.7088	19.7533	**19.7608**
		Std	0.0208	0.1092	0.0250	0.0839	0.0288	0.0543	0.7309	0.0274	**0.0108**
	6	Ave	22.5632	22.5106	22.5948	22.4892	22.5925	**22.6181**	20.8585	22.5662	22.5968
		Std	0.0438	0.2163	0.0785	0.2126	0.0840	0.1215	0.6742	0.0509	**0.0183**
	8	Ave	24.8474	24.6844	**24.9714**	24.6951	24.8048	24.7708	22.5335	24.9148	24.9555
		Std	0.1654	0.3198	0.1301	0.2041	0.2146	0.3173	0.8366	0.1127	**0.0767**
	10	Ave	26.4423	26.3396	26.6252	26.2922	26.5727	26.3547	23.9678	26.6752	**26.7027**
		Std	0.3312	0.3540	0.2607	0.2295	0.2625	0.3622	0.5838	0.1145	**0.0768**
Face	4	Ave	21.9753	21.9748	21.9903	21.9927	21.9776	**21.9991**	20.8896	21.9987	21.9887
		Std	0.0580	0.0489	0.0228	0.2058	0.0457	0.0870	1.0400	0.0819	**0.0162**
	6	Ave	24.4293	24.4617	24.4848	24.3839	**24.5437**	24.5141	22.7836	24.4756	24.5129
		Std	0.1590	0.1900	0.1805	0.2623	0.2033	0.2657	0.9994	0.1899	**0.1320**
	8	Ave	**26.4981**	26.2963	26.4884	26.0760	26.4107	26.4121	24.4856	26.4245	26.4580
		Std	0.1228	0.2323	0.1279	0.3891	0.2592	0.2162	0.9382	0.1472	**0.0596**
	10	Ave	28.2714	28.0357	28.0752	27.8417	28.0789	28.1672	25.0484	28.1907	**28.2848**
		Std	0.1709	0.4299	0.3364	0.3538	0.3156	0.2083	1.2902	0.2676	**0.0814**
Peppers	4	Ave	20.4512	20.4242	20.3952	20.4121	20.4402	20.3999	19.3902	20.4490	**20.4568**
		Std	0.0146	0.0801	0.1818	0.0780	0.0514	0.1693	0.6703	0.0170	**0.0094**
	6	Ave	23.2206	23.1763	23.2238	23.0795	23.2137	23.2000	21.6330	23.2158	**23.2275**
		Std	0.0645	0.1322	0.0237	0.1322	0.0427	0.1006	0.5357	0.0378	**0.0011**
	8	Ave	24.9516	24.8590	24.9441	24.6881	24.8788	24.9324	22.8329	24.9319	**24.9552**
		Std	0.0304	0.1597	0.0828	0.1793	0.1286	0.1790	0.5099	0.0358	**0.0094**
	10	Ave	26.4433	26.4724	26.6462	26.2300	26.4273	26.4412	24.0182	26.5766	**26.6973**
		Std	0.2465	0.2879	0.1813	0.2355	0.3053	0.2250	0.7351	0.1885	0.1377
Saturn	4	Ave	22.3356	22.3081	22.3270	22.3222	22.3305	**22.3461**	21.5054	22.3352	22.3256
		Std	0.0185	0.2183	0.0079	0.0732	0.0242	0.0203	0.7460	0.0223	**0.0000**
	6	Ave	25.3744	25.1549	25.3205	25.2432	25.3382	25.3393	23.6710	**25.3821**	25.3770
		Std	0.0234	0.2847	0.2220	0.1332	0.1588	0.0537	0.7864	0.0304	**0.0052**
	8	Ave	27.5085	27.3337	27.4633	27.2656	27.4701	27.4682	25.5531	27.5164	**27.5189**
		Std	0.0741	0.2714	0.1698	0.1848	0.1344	0.0964	0.8023	0.0669	**0.0269**
	10	Ave	**29.2325**	29.0452	29.0719	28.8716	29.0741	29.1537	26.6517	29.1974	29.2305
Friedman-rank			4.45	4.80	4.39	6.25	4.75	4.34	8.78	3.85	**3.39**
Final-rank			5	7	4	8	6	3	9	2	**1**

**Table 12:** Results of the FSIM values using Otsu’s method.

Images	TH	Metrics	VPPSO	IAGWO	IWOA	MadDE	SBO	AOO	HSO	RIME	MSRIME
Brian	4	Ave	0.6751	0.6751	0.6751	0.6748	0.6750	0.6750	0.6697	0.6749	**0.6752**
		Std	**0.0001**	0.0002	0.0002	0.0006	0.0002	0.0002	0.0318	0.0001	0.0004
	6	Ave	0.7529	0.7534	0.7681	0.7751	0.7912	0.8147	0.6778	0.7767	**0.9030**
		Std	0.1015	0.0999	0.1083	0.0572	0.1146	0.1159	0.1110	0.1118	**0.0059**
	8	Ave	0.9137	0.9479	0.9402	0.9457	0.9485	0.9482	0.7741	0.9459	**0.9502**
		Std	0.0841	0.0063	0.0454	0.0032	0.0042	0.0020	0.0984	0.0146	**0.0017**
	10	Ave	0.9612	0.9623	0.9626	0.9593	0.9608	0.9619	0.7897	0.9635	**0.9646**
		Std	0.0032	0.0037	0.0034	0.0029	0.0055	0.0029	0.1075	0.0026	**0.0018**
Camera	4	Ave	0.8353	0.8352	0.8335	0.8318	0.8359	0.8351	0.8210	0.8349	**0.8364**
		Std	0.0057	0.0060	0.0048	0.0042	0.0042	0.0054	0.0107	0.0046	**0.0022**
	6	Ave	0.8779	0.8761	**0.8788**	0.8751	0.8780	0.8774	0.8475	0.8779	0.8787
		Std	0.0029	0.0062	**0.0006**	0.0051	0.0015	0.0023	0.0124	0.0018	0.0007
	8	Ave	0.9018	0.9013	0.9022	0.8970	0.9015	0.9019	0.8638	0.9019	**0.9025**
		Std	0.0025	0.0039	0.0016	0.0057	0.0030	0.0027	0.0153	0.0026	**0.0011**
	10	Ave	0.9164	0.9160	0.9191	0.9118	0.9153	0.9166	0.8810	0.9185	**0.9193**
		Std	0.0043	0.0047	0.0015	0.0051	0.0061	0.0050	0.0174	0.0025	**0.0013**
Girl	4	Ave	0.7545	0.7536	0.7543	0.7529	0.7540	0.7536	0.7294	0.7542	**0.7547**
		Std	0.0008	0.0018	0.0009	0.0022	0.0013	0.0009	0.0192	0.0008	**0.0004**
	6	Ave	0.8435	0.8406	0.8438	0.8399	0.8434	0.8436	0.7893	0.8435	**0.8444**
		Std	0.0012	0.0075	0.0013	0.0040	0.0024	0.0022	0.0202	0.0013	**0.0005**
	8	Ave	0.8948	0.8891	0.8952	0.8867	0.8931	0.8896	0.8307	0.8954	**0.8966**
		Std	0.0016	0.0078	0.0014	0.0049	0.0042	0.0068	0.0203	0.0013	**0.0007**
	10	Ave	0.9230	0.9183	0.9241	0.9156	0.9222	0.9181	0.8586	0.9250	**0.9258**
		Std	0.0028	0.0063	0.0042	0.0043	0.0045	0.0076	0.0184	0.0021	**0.0009**
Face	4	Ave	0.8296	0.8298	0.8296	0.8295	0.8295	**0.8301**	0.8018	0.8299	0.8294
		Std	**0.0009**	0.0010	0.0011	0.0019	0.0015	0.0012	0.0158	0.0010	0.0009
	6	Ave	0.8672	0.8687	0.8688	**0.8712**	0.8693	0.8705	0.8364	0.8689	0.8675
		Std	**0.0039**	0.0059	0.0050	0.0055	0.0052	0.0052	0.0168	0.0052	0.0052
	8	Ave	0.9039	0.9031	0.9048	0.9009	0.9028	**0.9051**	0.8624	0.9040	0.9045
		Std	0.0014	0.0025	0.0014	0.0038	0.0030	0.0022	0.0188	0.0008	**0.0005**
	10	Ave	0.9287	0.9266	0.9276	0.9229	0.9259	0.9291	0.8734	0.9291	**0.9302**
		Std	0.0025	0.0050	0.0045	0.0041	0.0050	0.0012	0.0184	0.0016	**0.0005**
Peppers	4	Ave	**0.7868**	0.7863	0.7866	0.7860	0.7865	0.7864	0.7748	0.7866	0.7868
		Std	0.0003	0.0017	0.0003	0.0013	0.0009	0.0010	0.0088	0.0004	**0.0000**
	6	Ave	0.8493	0.8493	0.8491	0.8457	0.8489	0.8491	0.8161	**0.8495**	0.8492
		Std	0.0007	0.0018	0.0006	0.0031	0.0015	0.0011	0.0108	0.0010	**0.0001**
	8	Ave	**0.8864**	0.8850	0.8857	0.8806	0.8851	0.8840	0.8428	0.8860	0.8864
		Std	0.0007	0.0028	0.0025	0.0041	0.0031	0.0034	0.0093	0.0009	**0.0003**
	10	Ave	0.9122	0.9097	0.9128	0.9047	0.9101	0.9094	0.8648	0.9129	**0.9145**
		Std	0.0023	0.0055	0.0025	0.0030	0.0047	0.0037	0.0125	0.0020	**0.0007**
Saturn	4	Ave	0.8478	0.8477	0.8477	0.8478	0.8479	**0.8482**	0.8425	0.8479	0.8477
		Std	0.0003	0.0028	0.0001	0.0016	0.0004	0.0007	0.0117	0.0005	**0.0000**
	6	Ave	0.8835	0.8825	0.8834	0.8845	0.8841	**0.8845**	0.8675	0.8836	0.8836
		Std	0.0003	0.0029	0.0024	0.0033	0.0012	0.0021	0.0125	0.0008	**0.0001**
	8	Ave	0.9123	0.9127	0.9132	0.9107	0.9128	**0.9157**	0.8906	0.9138	0.9130
		Std	0.0016	0.0029	0.0037	0.0039	0.0025	0.0032	0.0119	0.0022	**0.0003**
	10	Ave	0.9341	0.9314	0.9323	0.9301	0.9324	0.9339	0.9044	0.9338	**0.9343**
Friedman-rank			4.28	4.79	4.68	5.51	4.58	5.18	8.10	4.47	**3.43**
Final-rank			2	6	5	8	4	7	9	3	**1**

Significant values are in bold.

**Table 13:** Results of the SSIM values using Otsu’s method.

Images	TH	Metrics	VPPSO	IAGWO	IWOA	MadDE	SBO	AOO	HSO	RIME	MSRIME
Brian	4	Ave	0.3767	0.3767	0.3768	0.3768	0.3767	0.3769	0.3631	0.3766	**0.3770**
		Std	**0.0003**	0.0005	0.0004	0.0008	0.0004	0.0004	0.0188	0.0005	0.0005
	6	Ave	0.4669	0.4831	0.4859	0.4876	0.5089	0.5464	0.3837	0.4934	**0.6752**
		Std	0.1103	0.1352	0.1237	0.0864	0.1272	0.1380	0.1176	0.1210	**0.0086**
	8	Ave	0.6563	0.7020	0.7345	0.7080	0.7247	0.7332	0.4847	0.7477	**0.7693**
		Std	0.1140	0.0602	0.0773	0.0602	0.0548	0.0638	0.1333	0.0420	**0.0282**
	10	Ave	0.7696	0.7699	**0.7883**	0.7761	0.7548	0.7823	0.4937	0.7750	0.7827
		Std	0.0484	0.0619	0.0488	0.0451	0.0642	0.0449	0.1217	0.0459	**0.0381**
Camera	4	Ave	0.7243	0.7182	0.7154	0.7519	0.7259	0.7184	0.6762	0.7294	**0.7544**
		Std	0.0402	0.0359	0.0367	0.0216	0.0361	0.0382	0.0393	0.0372	**0.0119**
	6	Ave	0.8029	0.8012	**0.8050**	0.8006	0.8041	0.8033	0.7319	0.8039	0.8047
		Std	0.0087	0.0103	**0.0012**	0.0112	0.0055	0.0051	0.0563	0.0039	0.0015
	8	Ave	0.8332	0.8330	0.8339	0.8221	0.8329	0.8318	0.7702	0.8321	**0.8369**
		Std	0.0063	0.0115	0.0052	0.0182	0.0092	0.0114	0.0521	0.0068	**0.0028**
	10	Ave	0.8531	0.8610	0.8619	0.8469	0.8532	**0.8623**	0.8008	0.8594	0.8615
		Std	0.0101	0.0120	0.0083	0.0181	0.0170	0.0126	0.0515	0.0074	**0.0068**
Girl	4	Ave	0.7075	0.7063	0.7073	0.7048	0.7069	0.7063	0.6800	0.7072	**0.7080**
		Std	0.0014	0.0037	0.0016	0.0041	0.0018	0.0021	0.0291	0.0016	**0.0007**
	6	Ave	0.7997	0.7970	0.8002	0.7955	0.7996	0.8000	0.7490	0.7998	**0.8011**
		Std	0.0016	0.0070	0.0016	0.0062	0.0030	0.0021	0.0258	0.0017	**0.0007**
	8	Ave	0.8573	0.8524	0.8579	0.8499	0.8557	0.8525	0.7933	0.8578	**0.8590**
		Std	0.0018	0.0072	0.0016	0.0065	0.0044	0.0068	0.0243	0.0014	**0.0006**
	10	Ave	0.8921	0.8871	0.8922	0.8835	0.8908	0.8856	0.8258	0.8937	**0.8947**
		Std	0.0033	0.0066	0.0048	0.0048	0.0050	0.0090	0.0196	0.0027	**0.0009**
Face	4	Ave	0.7139	0.7141	0.7139	0.7144	0.7141	**0.7146**	0.6735	0.7143	0.7137
		Std	0.0008	0.0009	0.0009	0.0018	0.0010	0.0011	0.0296	0.0008	**0.0008**
	6	Ave	0.7531	0.7586	0.7571	0.7561	0.7591	0.7609	0.7233	0.7583	**0.7650**
		Std	**0.0113**	0.0161	0.0149	0.0194	0.0146	0.0159	0.0282	0.0157	0.0139
	8	Ave	0.8040	0.8082	0.8063	0.8041	0.8026	**0.8093**	0.7524	0.8050	0.8056
		Std	0.0059	0.0073	0.0051	0.0119	0.0074	0.0112	0.0293	0.0031	**0.0014**
	10	Ave	0.8342	0.8412	0.8395	0.8367	0.8346	0.8406	0.7665	0.8389	**0.8431**
		Std	0.0089	0.0113	0.0112	0.0163	0.0144	0.0111	0.0254	0.0044	**0.0043**
Peppers	4	Ave	**0.7141**	0.7135	0.7116	0.7137	0.7135	0.7125	0.6875	0.7140	0.7137
		Std	0.0010	0.0061	0.0062	0.0035	0.0028	0.0059	0.0177	0.0011	**0.0004**
	6	Ave	0.7866	**0.7872**	0.7865	0.7790	0.7861	0.7859	0.7453	0.7870	0.7869
		Std	0.0027	0.0026	0.0025	0.0067	0.0021	0.0034	0.0138	0.0020	**0.0001**
	8	Ave	0.8192	0.8190	0.8199	0.8148	0.8183	**0.8209**	0.7757	0.8182	0.8191
		Std	0.0011	0.0046	0.0032	0.0066	0.0042	0.0049	0.0183	0.0016	**0.0003**
	10	Ave	0.8508	0.8533	0.8555	0.8494	0.8513	0.8558	0.8062	0.8533	**0.8559**
		Std	0.0052	0.0075	0.0055	0.0082	0.0072	0.0079	0.0186	**0.0045**	0.0046
Saturn	4	Ave	0.8308	0.8304	0.8307	0.8309	0.8309	**0.8314**	0.8189	0.8309	0.8307
		Std	0.0002	0.0050	0.0001	0.0024	0.0006	0.0012	0.0178	0.0008	**0.0000**
	6	Ave	0.8798	0.8766	0.8788	0.8796	0.8800	0.8802	0.8540	0.8798	**0.8802**
		Std	0.0005	0.0065	0.0054	0.0033	0.0015	0.0027	0.0136	0.0009	**0.0001**
	8	Ave	0.9076	0.9062	0.9076	0.9056	0.9075	**0.9096**	0.8820	0.9088	0.9085
		Std	0.0015	0.0043	0.0045	0.0030	0.0021	0.0024	0.0146	0.0014	**0.0002**
	10	Ave	0.9284	0.9259	0.9268	0.9251	0.9269	**0.9288**	0.8986	0.9286	**0.9288**
Friedman-rank			4.40	5.02	4.52	5.60	4.78	4.68	8.35	4.29	**3.38**
Final-rank			3	7	4	8	6	5	9	2	**1**

Significant values are in bold.

The experiments consider four thresholding scenarios with 4, 6, 8, and 10 levels, all employing the Otsu criterion to search for the optimal thresholds. The results indicate that MSRIME consistently achieves superior performance across all metrics and threshold levels, highlighting its effectiveness and robustness in multi-level image segmentation tasks.

Table 10 reports the Otsu objective function values for six benchmark images under four threshold levels (TH = 4, 6, 8, 10). MSRIME consistently achieves the optimal or near-optimal values across all images and threshold levels, with its advantage becoming more pronounced as the number of thresholds increases. For instance, in the peppers image with TH = 10, MSRIME attains an average objective function value of 2.8086E+03, which represents a 0.068% improvement over the original RIME (2.8067E+03) and a 1.01% improvement over HSO (2.7804E+03), indicating its ability to maximize inter-class variance through more precise threshold combinations. In the brain image (low-contrast scenario) with TH = 8, MSRIME achieves 3.7636E+03, higher than RIME−S3 (3.7631E+03) and SBO (3.7630E+03), demonstrating its capability for fine segmentation of low-contrast images.

From the perspective of evaluation metrics, Tables 11, 12 and 13 further confirm MSRIME’s superiority in segmentation quality. In the camera image with TH = 4, MSRIME achieves a PSNR of 19.7868 dB, representing a 3.5% improvement over the original RIME (19.1182 dB) and a 4.1% improvement over VPPSO (19.0010 dB), indicating significantly reduced image distortion. In the girl image with TH = 10, MSRIME attains an FSIM of 0.9258, outperforming RIME (0.9250) and IAGWO (0.9183), reflecting superior feature preservation. In the face image with TH = 8, MSRIME achieves an SSIM of 0.8056, close to 1, and 7.1% higher than HSO (0.7524), indicating a better structural similarity between the segmented and original images.

The standard deviation (Std) values highlight the stability of the segmentation results. MSRIME exhibits consistently lower variability across all metrics. In Table 9, for the saturn image with TH = 4, MSRIME’s objective function Std is 9.2504E−13, nearly zero, which is 99.99% lower than RIME (2.6045E−02) and 99.997% lower than MadDE (3.2307E−01). In Table 10, for the peppers image with TH = 6, the PSNR Std of MSRIME is only 0.0011 dB, far below RIME (0.0378 dB) and IWOA (0.0237 dB), demonstrating stable image quality across multiple runs.

At higher threshold levels (TH = 10), the stability advantage of MSRIME becomes even more pronounced. In Table 11, for the brain image, MSRIME’s FSIM Std is 0.0018, lower than RIME (0.0026) and SBO (0.0055). In Table 12, for the camera image, MSRIME’s SSIM Std is 0.0068, only 56.7% of IAGWO’s 0.0120, further confirming its robustness in complex segmentation tasks. This enhanced stability can be attributed to the algorithm’s strategy probability self-adaptive selection mechanism, which dynamically adjusts the search strategies to reduce random fluctuations during optimization.

**Fig. 8:**
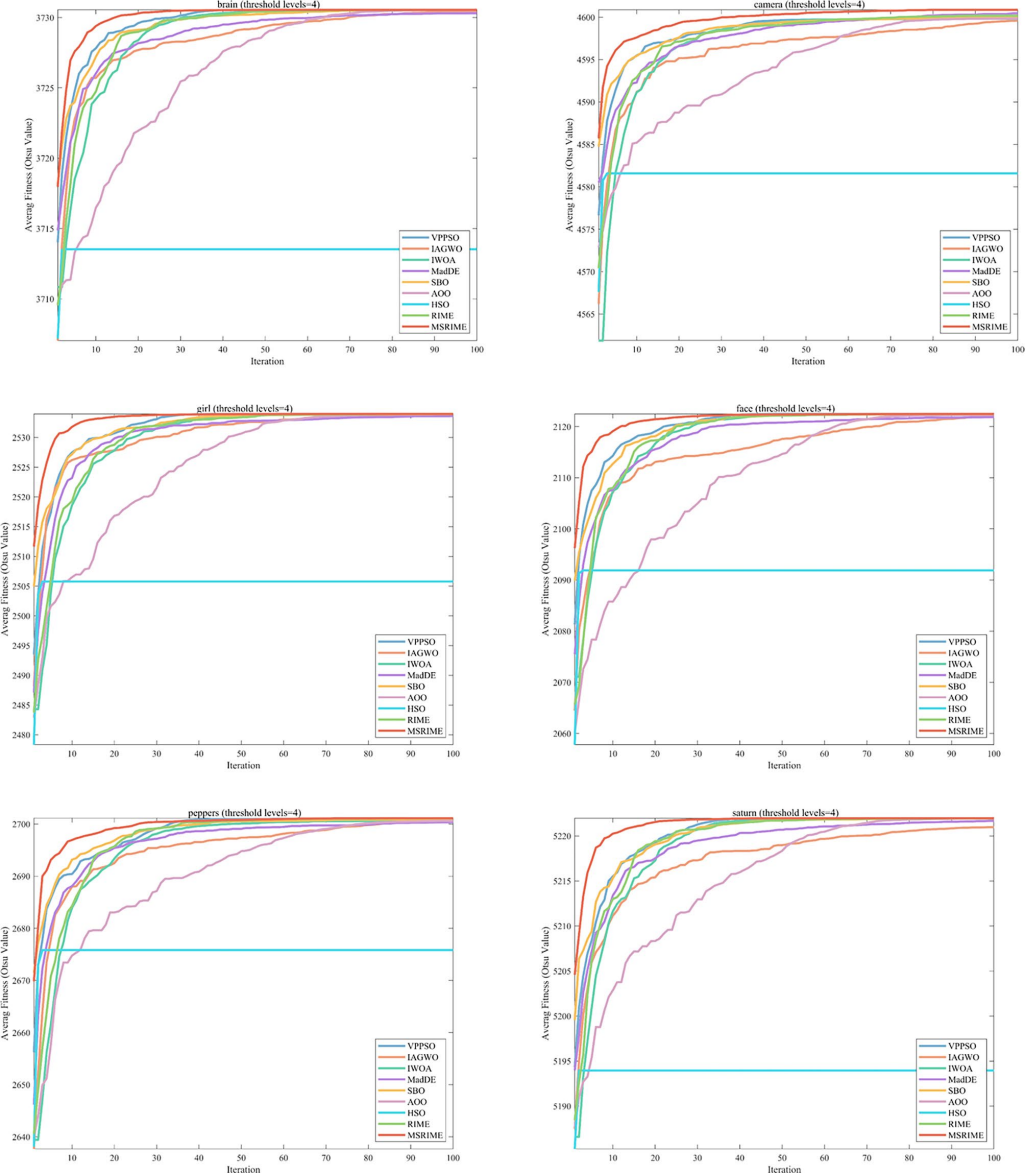
Fitness value curves of different algorithms across various images (TH = 4).

Figure 8 (fitness convergence curves, TH = 4) intuitively illustrates the high convergence efficiency of MSRIME. For images such as brain, camera, and face, MSRIME consistently achieves the highest fitness values (objective function values) and converges faster than all comparative algorithms. For example, in the camera image, MSRIME reaches stable convergence at iteration 30 (fitness ≈ 4.6009E+03), whereas the original RIME requires over 60 iterations to stabilize and still attains a lower final fitness. In the peppers image, MSRIME’s fitness rapidly increases during the initial 20 iterations, quickly surpassing other algorithms and maintaining stability in later iterations, avoiding convergence stagnation. This behavior benefits from the dynamic adjustment of the self-adaptive differential mutation factor, which enhances global exploration in the early stage and focuses on local exploitation in the later stage.

Comparing the convergence curves across different images, MSRIME demonstrates robust adaptability to varying image characteristics—low contrast (brain), high brightness (camera), and complex textures (peppers)—with smooth convergence curves and minimal fluctuation. In contrast, HSO, IWOA, and other algorithms exhibit convergence oscillations on complex images such as saturn, further confirming the stability and reliability of MSRIME’s dynamic optimization process.

**Fig. 9:**
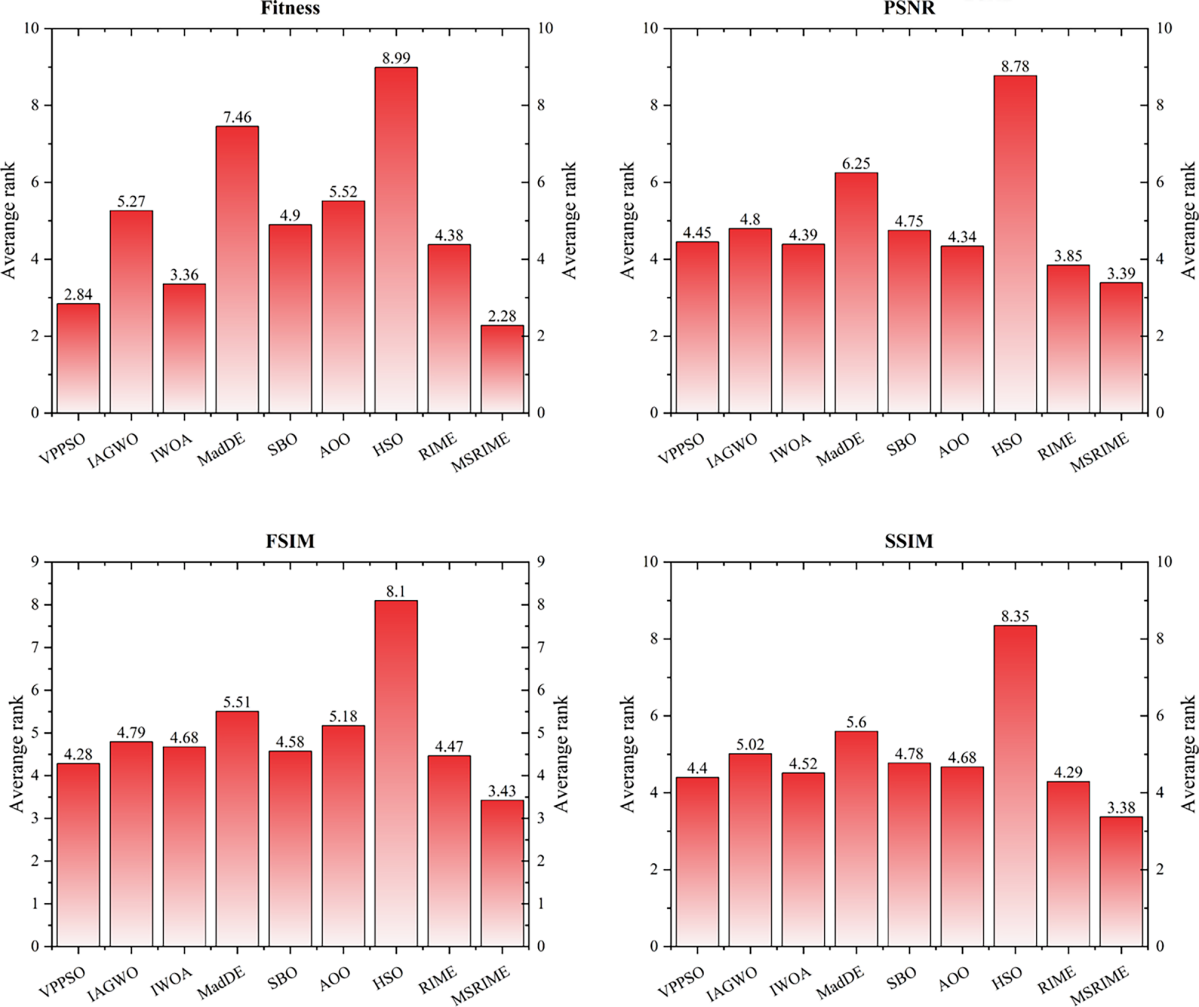
Average rankings of each algorithm across different metrics.

Figure 9 (heatmap of average rankings across multiple metrics) quantifies the overall advantage of MSRIME from a global perspective. Across the four core metrics—objective function value, PSNR, FSIM, and SSIM—MSRIME consistently achieves the top average ranking, with significant gaps over other algorithms. In objective function ranking, MSRIME attains an average rank of 2.28, only 52.1% of the second-ranked RIME (4.38). For PSNR, MSRIME leads with an average rank of 3.39, ahead of RIME (3.85). In FSIM and SSIM rankings, MSRIME holds first place with average ranks of 3.43 and 3.38, outperforming the lowest-ranked HSO (8.78 and 8.35) by more than five ranking units.

From an overall performance perspective, MSRIME’s rankings are concentrated in the “excellent” range across all metrics, showing no obvious weaknesses. In contrast, other algorithms exhibit uneven performance: MadDE ranks relatively well in PSNR (6.25) but poorly in objective function values (7.46), while SBO performs strongly in low-threshold scenarios but drops in high-threshold segmentation. This demonstrates that MSRIME’s multi-strategy fusion design achieves a balanced optimization of segmentation accuracy, stability, and convergence efficiency, making it a highly effective solution for multi-level threshold image segmentation tasks.

In summary, the quantitative data in Tables 9, 10, 11 and 12 combined with the visual results in Figs. 6 and 7 confirm that MSRIME, based on the Otsu criterion, can precisely optimize the objective function, enhance segmentation quality, maintain stable performance, and achieve fast convergence. Its overall superiority over the original RIME and other mainstream optimization algorithms provides reliable technical support for fine-grained segmentation in applications such as medical imaging and remote sensing.

## Conclusion and future work

This study addresses the limitations of the traditional RIME algorithm in global optimization and multi-level threshold image segmentation tasks, including imbalanced exploration–exploitation, poor parameter adaptability, and single-strategy design. To overcome these issues, we propose a Multi-Strategy Self-Adaptive RIME Optimization algorithm (MSRIME) and validate its superior performance through systematic experiments. In terms of algorithm design, MSRIME achieves breakthroughs through three core improvements: the introduction of an adaptive differential mutation factor adjustment strategy, which dynamically tunes the mutation factor *F* based on the iteration process to balance global exploration and local exploitation; the construction of a multi-strategy collaborative update pool, integrating the standard RIME strategy, DE/rand/1, DE/current-to-best/1, and a random-weight guided differential strategy to cover the entire optimization cycle; and the design of a self-adaptive strategy selection mechanism, which quantifies strategy effectiveness via a performance counter and dynamically adjusts selection probability through a roulette-wheel algorithm, enabling adaptive alignment between optimization progress and strategy selection.

In numerical experiments on the CEC2017 (30D, 100D) and CEC2022 (10D, 20D) benchmark function sets, MSRIME consistently outperforms the original RIME as well as eight mainstream algorithms including VPPSO and IAGWO. Friedman test results indicate that MSRIME ranks first across all test scenarios, achieving the lowest average ranking (1.67–2.17), and exhibits the smallest performance degradation in high-dimensional environments. For example, on the CEC2017-30D unimodal function F1, the average fitness of MSRIME is only 0.13% of that of the original RIME; on the multimodal function F9, optimization accuracy improves by 51.28% while the standard deviation decreases by 43.5%, demonstrating excellent optimization precision and stability.

For practical application validation, using Otsu’s maximum between-class variance as the objective function, 4–10 level threshold segmentation experiments were conducted on six benchmark images including brain, camera, and peppers. MSRIME achieves the best performance across four metrics: objective function value, PSNR, FSIM, and SSIM. For instance, in 4-level threshold segmentation of the camera image, PSNR reaches 19.7868 dB, a 3.5% improvement over the original RIME; in 10-level threshold segmentation of the peppers image, FSIM and SSIM approach theoretical optima, with standard deviation only 10–50% of that of comparative algorithms, fully demonstrating its practical value in real image processing scenarios.

Despite its effectiveness, the proposed MSRIME algorithm still has some limitations. First, the integration of multiple strategies increases algorithmic complexity compared to the original RIME. In high-dimensional scenarios, multi-strategy collaboration and probability selection introduce additional computational overhead, with single-iteration time increasing by approximately 15–% at 100 dimensions, which may limit its applicability in large-scale real-time problems. Second, although the adaptive mechanism improves robustness, it may introduce additional computational burden and does not fully consider local features of the optimization landscape, leading to potential delays in strategy switching in dense extrema regions of multimodal functions. Third, the current study focuses primarily on continuous optimization and image segmentation tasks; its performance on discrete or combinatorial optimization problems requires further investigation. In addition, core parameters still require manual initialization, and parameter fluctuations may cause performance variations of approximately 8%–12%.

Future work will focus on addressing these limitations. Specifically, we will explore lightweight strategy selection mechanisms and strategy pruning to improve computational efficiency, as well as parallel computation to accelerate high-dimensional optimization. Furthermore, problem-feature-aware modules and reinforcement learning will be integrated into the strategy selection framework to enhance adaptability. The proposed method will also be extended to broader application domains, such as robot path planning and medical image analysis, and a “scenario–strategy” mapping mechanism will be designed to improve generalization capability. In addition, parameter recommendation models and perturbation testing strategies will be developed to reduce parameter sensitivity. Finally, hybridization with deep learning and evolutionary frameworks will be investigated to further enhance performance in complex optimization scenarios.

## Data Availability

The original contributions presented in this study are included in the article. Further inquiries can be directed to the corresponding author.
